# Supplementary figures and images for: Prevalence of dermatological toxicities in patients with melanoma undergoing immunotherapy: Systematic review and meta-analysis
Source: PLoS One. 2021 Aug 6;16(8):e0255716. doi: 10.1371/journal.pone.0255716 (PMC8345892; doi:10.1371/journal.pone.0255716)

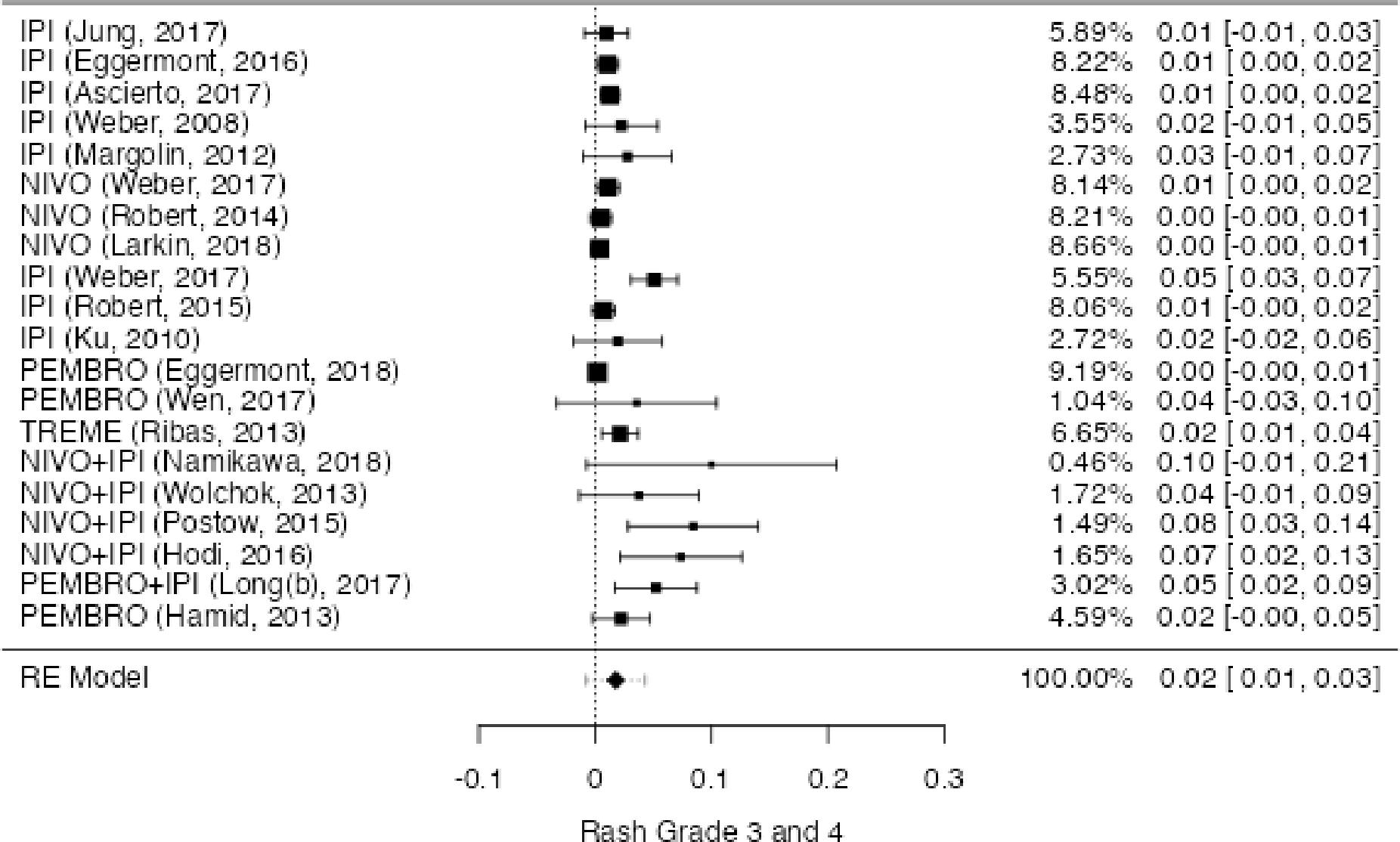

Supplement: S1 Fig — (TIF) [file pone.0255716.s002.tif]

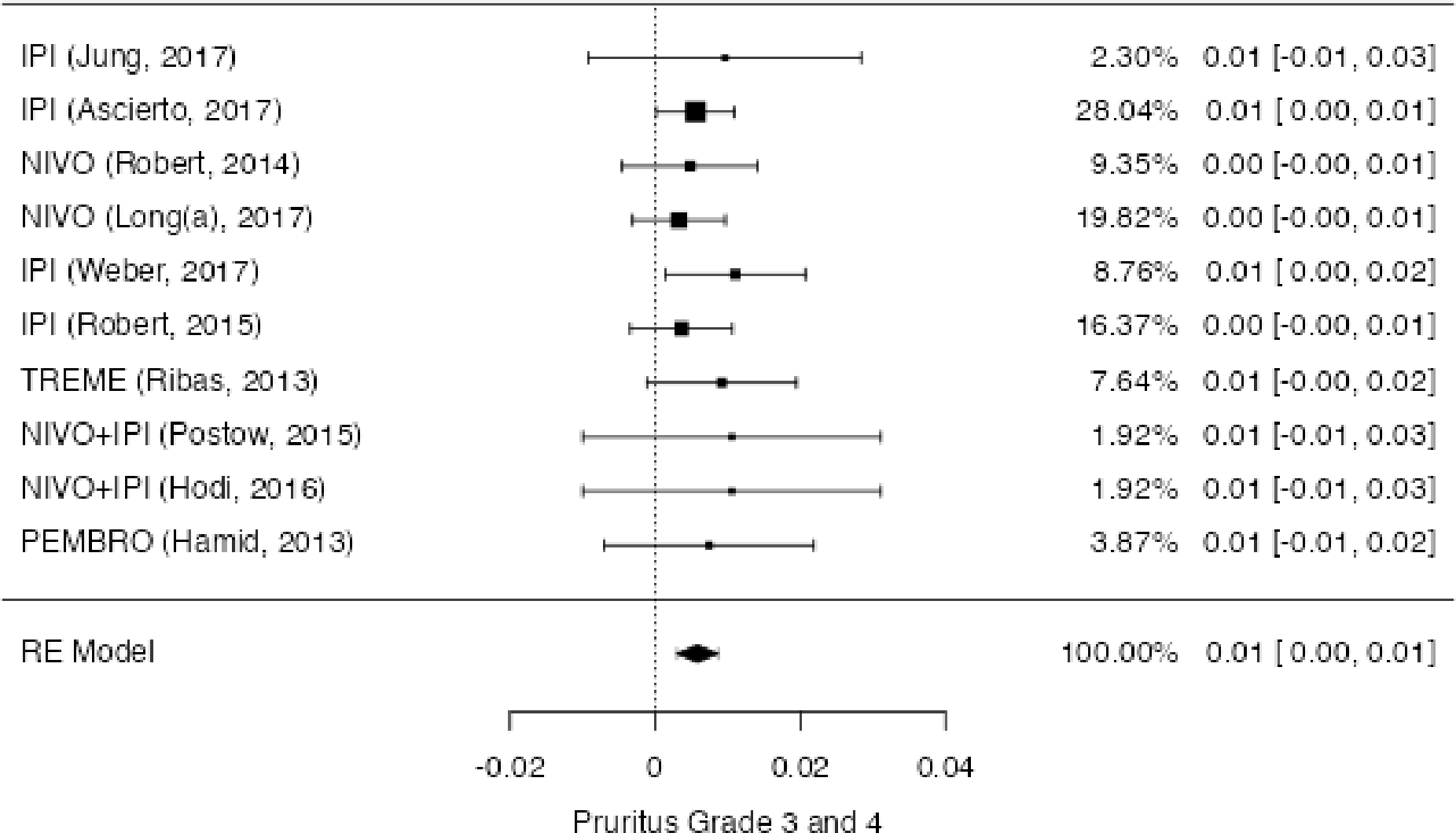

Supplement: S2 Fig — (TIF) [file pone.0255716.s003.tif]
