# Supplementary material for: Prevalence of dermatological toxicities in patients with melanoma undergoing immunotherapy: Systematic review and meta-analysis
Source: PLoS One. 2021 Aug 6;16(8):e0255716. doi: 10.1371/journal.pone.0255716 (PMC8345892; doi:10.1371/journal.pone.0255716)
Supplement: S1 File — (DOCX) [file pone.0255716.s004.docx]

**S1 File** - Search Strategy performed in databases CINAHL, COCHRANE CENTRAL, LILACS, LIVIVO, PUBMED, SCOPUS, WEB OF SCIENCE, GOOGLE SCHOLAR and OPENGRAY on January 23^th^, 2019.

**CINAHL**

| **Search Strategy** | **Results** |
| --- | --- |
| Search (S1 and S2 and S3) | 192 |
| (("Neoplasms" OR "Neoplasm" OR "cancer" OR "Tumors" OR "Tumor" OR "Cancer" OR "Cancers" OR "Malignant Neoplasms" OR "Malignant Neoplasm") AND ("immune checkpoint" OR "checkpoint inhibitors" OR "checkpoint inhibitor" OR "Antineoplastic Agents, Immunological" OR "Immunological Antineoplastic Agents" OR "Anti-CTLA-4 agents" OR "anti-PD1 agents" OR "Immune therapy" OR "Immunotherapy" OR "Ipilimumab" OR "checkpoint inhibitors" OR "pembrolizumab" OR "nivolumab" OR "immune-checkpoint" OR "PD-1" OR "PD-L1" OR "atezolizumab" OR "immune checkpoint inhibitor" OR "immune checkpoint inhibitors" OR "tremelimumab" OR "durvalumab" OR "anti-PD-1 antibodies" OR "avelumab" OR "pidilizumab" OR "anti-CTLA-4" OR "anti-PD-1" OR "anti-PD-L1" OR "anti-CTLA4 antibodies" OR "Anti-CTLA-4 agents" OR "anti-PD1 agents" OR "anti-PD-L1 agents" OR "CTLA-4-blocking antibody" OR "lambrolizumab") AND ("cutaneous side effects" OR "cutaneous adverse reactions" OR "skin toxicity" OR "skin toxicity events" OR "skin manifestations" OR "cutaneous reactions" OR "cutaneous reaction" OR "Manifestation Skin" OR "Manifestations Skin" OR "Skin Manifestation" OR "Skin Manifestations” OR "Drug eruptions" OR "Drug Eruption" OR "Eruption Drug" OR "dermatological reaction" OR "dermatological reactions" OR "skin-related toxicities" OR "cutaneous adverse effects" OR "cutaneous adverse effect" OR "dermatologic toxicities" OR "dermatologic toxicity" OR "rash" OR "skin rash" OR "skin rashes" OR "pruritus" OR "vitiligo" OR "drug reaction" OR "macular eruption" OR "papular eruption" OR "maculopapular eruption" OR "skin eruption" OR "erythema" OR "desquamation" OR "erythroderma" OR "vesicular eruption" OR "exfoliative dermatitis" OR "ulcerative dermatitis" OR "bullous dermatitis" OR "dermatitis" OR "eczema" OR "skin-related toxicity" OR "lichenoid dermatitis" OR "xerosis" OR "alopecia" OR "urticaria" OR "photosensitivity reaction" OR "hyperhidrosis" OR "skin exfoliation" OR "hair color changes" OR "maculopapular exanthema" OR "rosacea" OR "pyoderma gangrenosum" OR "acneiform rash" OR "cutaneous sarcoidosis" OR "dermatomyositis" OR "DRESS syndrome" OR "Stevens Johnson syndrome" OR "toxic epidermal necrolysis" OR "toxic epidermal necrosis" OR "psoriasiform eruption" OR "mucositis" OR "hand-foot syndrome" OR "hypopigmentation" OR "sweet syndrome" OR "Grover disease" OR "hair depigmentation" OR "psoriasis" OR "bullous pemphigoid" OR "alopecia areata" OR "lichenoid reactions" OR "vasculitis" OR "cellulitis" OR "sclerodermoid reaction" OR "cutaneous adverse effects" OR "actinic keratoses" OR "squamous cell carcinoma" OR "seborrheic keratoses" OR "lichen planus" OR "hair growth" OR "hyperkeratosis" OR "atopic dermatitis" OR "stomatitis" OR "dermatitis herpetiformis" OR "oral lichenoid reaction" OR "Dysgeusia" OR "ulcerations" OR "rash maculopapular" OR "erythematous rash" OR "pruritic rash" OR "drug eruption" OR "papules" OR "plaques" OR "vitiligo-like" OR "depigmentation" OR "pruritic maculopapular rash")) |  |

**COCHRANE CENTRAL**

| **Search Strategy** | **Results** |
| --- | --- |
| #1 and #2 and #3  (("Neoplasms" OR "Neoplasm" OR "cancer" OR "Tumors” OR "Tumor" OR "Cancer" OR "Cancers" OR "Malignant Neoplasms" OR "Malignant Neoplasm")):ti,ab,kw AND (("immune checkpoint" OR "checkpoint inhibitors" OR "checkpoint inhibitor" OR "Antineoplastic Agents, Immunological" OR "Immunological Antineoplastic Agents" OR "Anti-CTLA-4 agents" OR "anti-PD1 agents" OR “Immune therapy” OR "Immunotherapy" OR "Ipilimumab" OR "checkpoint inhibitors" OR "pembrolizumab” OR "nivolumab" OR "PD-1" OR "PD-L1" OR "atezolizumab" OR "immune checkpoint inhibitor" OR "immune checkpoint inhibitors" OR "tremelimumab" OR "durvalumab" OR "anti-PD-1 antibodies" OR "avelumab" OR "pidilizumab" OR "anti-CTLA-4" OR "anti-PD-1" OR "anti-PD-L1" OR "anti-CTLA4 antibodies" OR "Anti-CTLA4 agents" OR "anti-PD1 agents" OR "anti-PD-L1 agents" OR "CTLA-4–blocking antibody" OR "lambrolizumab")):ti,ab,kw AND ((“cutaneous side effects” OR “cutaneous adverse reactions” OR “skin toxicity” OR “skin toxicity events” OR “skin manifestations” OR “cutaneous reactions” OR “cutaneous reaction” OR “Manifestation Skin” OR “Manifestations Skin” OR “Skin Manifestation” OR “Skin Manifestations” OR “Drug eruptions” OR “Drug Eruption” OR “Eruption Drug” OR “dermatological reaction” OR “dermatological reactions” OR “skin-related toxicities” OR “cutaneous adverse effects” OR “cutaneous adverse effect” OR “dermatologic toxicities” OR “dermatologic toxicity” OR "rash" OR "skin rash" OR "skin rashes" OR "pruritus" OR "vitiligo" OR "drug reaction" OR "macular eruption" OR "papular eruption" OR "maculopapular eruption" OR "skin eruption" OR "erythema" OR "desquamation" OR "erythroderma" OR "vesicular eruption" OR "exfoliative dermatitis" OR "ulcerative dermatitis" OR "bullous dermatitis" OR "dermatitis" OR "eczema" OR "skin-related toxicity" OR "lichenoid dermatitis" OR "xerosis" OR "alopecia" OR "urticaria" OR "photosensitivity reaction" OR "hyperhidrosis" OR "skin exfoliation" OR "hair color changes" OR "maculopapular exanthema" OR "rosacea" OR "pyoderma gangrenosum" OR "acneiform rash" OR "cutaneous sarcoidosis" OR "dermatomyositis" OR "DRESS syndrome" OR "Stevens Johnson syndrome" OR "toxic epidermal necrolysis" OR "psoriasiform eruption" OR "mucositis" OR "hand-foot syndrome" OR "hypopigmentation" OR "sweet syndrome" OR "Grover disease" OR "hair depigmentation" OR "psoriasis" OR "bullous pemphigoid" OR "alopecia areata" OR "lichenoid reactions" OR "vasculitis" OR "cellulitis" OR "sclerodermoid reaction" OR "cutaneous adverse effects" OR "actinic keratoses" OR "squamous cell carcinoma" OR "seborrheic keratoses" OR "lichen planus" OR "hair growth" OR "hyperkeratosis" OR "atopic dermatitis" OR "stomatitis" OR "dermatitis herpetiformis" OR "oral lichenoid reaction" OR "Dysgeusia" OR "ulcerations" OR "rash maculopapular" OR "erythematous rash" OR "pruritic rash" OR "drug eruption" OR "papules" OR "plaques" OR "vitiligo-like" OR "depigmentation" OR "pruritic maculopapular rash")) in Title, Abstracts and keywords in Trials (Word variations have been searched) | 1,269 |
|  |  |

**LILACS**

| **Search Strategy** | **Results** |
| --- | --- |
| (tw:((" "Neoplasms" OR "Neoplasias" OR "Neoplasias" AND "Immunotherapy" OR "Inmunoterapia" OR "Imunoterapia" OR "Antineoplastic Agents, Immunological" OR "Antineoplásicos Inmunológicos" OR "Antineoplásicos Imunológicos" AND "Skin Manifestations" OR "Manifestaciones Cutáneas" OR "Manifestações Cutâneas" OR "Drug Eruptions" OR "Erupciones por Medicamentos" OR "Erupção por Droga" OR "Skin Manifestations" OR "Manifestaciones Cutáneas" OR "Manifestações Cutâneas")) | 0 |

**LIVIVO**

| **Search Strategy** | **Results** |
| --- | --- |
| Cancer, Immune checkpoint inhibitors, Skin-related toxicities | 77 |

**PUBMED**

| **Search Strategy** | **Results** |
| --- | --- |
| (#1 AND #2 AND #3) | 3,304 |
| (((("Neoplasms"[Mesh] OR "Neoplasm" OR "cancer" OR "Tumors" OR "Tumor" OR "Cancer" OR "Cancers" OR "Malignant Neoplasms" OR "Malignant Neoplasm"))) AND (("immune checkpoint" OR "checkpoint inhibitors" OR "checkpoint inhibitor" OR "Antineoplastic Agents, Immunological"[Mesh] OR "Immunological Antineoplastic Agents" OR "Anti-CTLA-4 agents" OR "anti-PD1 agents" OR "Immune therapy" OR "Immunotherapy" OR "Ipilimumab" OR "checkpoint inhibitors" OR "pembrolizumab" OR "nivolumab" OR "immune-checkpoint" OR "PD-1" OR "PD-L1" OR "atezolizumab" OR "immune checkpoint inhibitor" OR "immune checkpoint inhibitors" OR "tremelimumab" OR "durvalumab" OR "anti-PD-1 antibodies" OR "avelumab" OR "pidilizumab" OR "anti-CTLA-4" OR "anti-PD-1" OR "anti-PD-L1" OR "anti-CTLA4 antibodies" OR "Anti-CTLA-4 agents" OR "anti-PD1 agents" OR "anti-PD-L1 agents" OR "CTLA-4-blocking antibody" OR "lambrolizumab"))) AND (("cutaneous side effects" OR "cutaneous adverse reactions" OR "skin toxicity" OR "skin toxicity events" OR "skin manifestations" OR "cutaneous reactions" OR "cutaneous reaction" OR "Manifestation Skin" OR "Manifestations Skin" OR "Skin Manifestation" OR "Skin Manifestations"[Mesh Terms] OR "Drug eruptions" [Mesh Terms] OR "Drug Eruption" OR "Eruption Drug" OR "dermatological reaction" OR "dermatological reactions" OR "skin-related toxicities" OR "cutaneous adverse effects" OR "cutaneous adverse effect" OR "dermatologic toxicities" OR "dermatologic toxicity" OR "rash" OR "skin rash" OR "skin rashes" OR "pruritus" OR "vitiligo" OR "drug reaction" OR "macular eruption" OR "papular eruption" OR "maculopapular eruption" OR "skin eruption" OR "erythema" OR "desquamation" OR "erythroderma" OR "vesicular eruption" OR "exfoliative dermatitis" OR "ulcerative dermatitis" OR "bullous dermatitis" OR "dermatitis" OR "eczema" OR "skin-related toxicity" OR "lichenoid dermatitis" OR "xerosis" OR "alopecia" OR "urticaria" OR "photosensitivity reaction" OR "hyperhidrosis" OR "skin exfoliation" OR "hair color changes" OR "maculopapular exanthema" OR "rosacea" OR "pyoderma gangrenosum" OR "acneiform rash" OR "cutaneous sarcoidosis" OR "dermatomyositis" OR "DRESS syndrome" OR "Stevens Johnson syndrome" OR "toxic epidermal necrolysis" OR "toxic epidermal necrosis" OR "psoriasiform eruption" OR "hand-foot syndrome" OR "hypopigmentation" OR "sweet syndrome" OR "Grover disease" OR "hair depigmentation" OR "psoriasis" OR "bullous pemphigoid" OR "alopecia areata" OR "lichenoid reactions" OR "vasculitis" OR "cellulitis" OR "sclerodermoid reaction" OR "cutaneous adverse effects" OR "actinic keratoses" OR "squamous cell carcinoma" OR "seborrheic keratoses" OR "lichen planus" OR "hair growth" OR "hyperkeratosis" OR "atopic dermatitis" OR "stomatitis" OR "dermatitis herpetiformis" OR "oral lichenoid reaction" OR "ulcerations" OR "rash maculopapular" OR "erythematous rash" OR "pruritic rash" OR "drug eruption" OR "papules" OR "plaques" OR "vitiligo-like" OR "depigmentation" OR "pruritic maculopapular rash")) Sort by: Relevance |  |

**SCOPUS**

| **Search Strategy** | **Results** |
| --- | --- |
| #1 AND #2 AND #3 | 2,033 |
| TITLE-ABS-KEY ("Neoplasms" OR "Neoplasm" OR "cancer" OR "Tumors" OR "Tumor" OR "Cancer" OR "Cancers" OR "Malignant Neoplasms" OR "Malignant Neoplasm" AND "immune checkpoint" OR "checkpoint inhibitors" OR "checkpoint inhibitor" OR "Antineoplastic Agents, Immunological" OR "Immunological Antineoplastic Agents" OR "Anti-CTLA-4 agents" OR "anti-PD1 agents" OR "Immune therapy" OR "Immunotherapy" OR "Ipilimumab" OR "checkpoint inhibitors" OR "pembrolizumab" OR "nivolumab" OR "immune-checkpoint" OR "PD-1" OR "PD-L1" OR "atezolizumab" OR "immune checkpoint inhibitor" OR "immune checkpoint inhibitors" OR "tremelimumab" OR "durvalumab" OR "anti-PD-1 antibodies" OR "avelumab" OR "pidilizumab" OR "anti-CTLA-4" OR "anti-PD-1" OR "anti-PD-L1" OR "anti-CTLA4 antibodies" OR "Anti-CTLA4 agents" OR "anti-PD1 agents" OR "anti-PD-L1 agents" OR "CTLA-4--blocking antibody" OR "lambrolizumab" AND "cutaneous side effects" OR "cutaneous adverse reactions" OR "skin toxicity" OR "skin toxicity events" OR "skin manifestations" OR "cutaneous reactions" OR "cutaneous reaction" OR "Manifestation Skin" OR "Manifestations Skin" OR "Skin Manifestation" OR "Skin Manifestations" OR "Drug eruptions" OR "Drug Eruption" OR "Eruption Drug" OR "dermatological reaction" OR "dermatological reactions" OR "skin-related toxicities" OR "cutaneous adverse effects" OR "cutaneous adverse effect" OR "dermatologic toxicities" OR "dermatologic Toxicity" AND ( EXCLUDE ( DOCTYPE , "re" ) OR EXCLUDE ( DOCTYPE , "ch" ) OR EXCLUDE ( DOCTYPE , "cp" ) OR EXCLUDE ( DOCTYPE , "le" ) OR EXCLUDE ( DOCTYPE , "sh" ) OR EXCLUDE ( DOCTYPE , "no" ) OR EXCLUDE ( DOCTYPE , "bk" ) OR EXCLUDE ( DOCTYPE , "ed" ) ) "ulcerations" OR "rash maculopapular" OR "erythematous rash" OR "pruritic rash" OR "drug eruption" OR "papules" OR "plaques" OR "vitiligo-like" OR "depigmentation" OR "pruritic maculopapular rash")) |  |

**WEB OF SCIENCE**

| **Search Strategy** | **Results** |
| --- | --- |
| #1 AND #2 AND #3 | 2,927 |
| Tópico: ("Neoplasms" OR "Neoplasm" OR "cancer" OR "Tumors” OR "Tumor" OR "Cancer" OR "Cancers" OR "Malignant Neoplasms" OR "Malignant Neoplasm") AND TOPIC:("immune checkpoint" OR "checkpoint inhibitors" OR "checkpoint inhibitor" OR "Antineoplastic Agents, Immunological" OR "Immunological Antineoplastic Agents" OR "Anti-CTLA-4 agents" OR "anti-PD1 agents" OR “Immune therapy” OR "Immunotherapy" OR "Ipilimumab" OR "checkpoint inhibitors" OR "pembrolizumab” OR "nivolumab" OR "immune-checkpoint" OR "PD-1" OR "PD-L1" OR "atezolizumab" OR "immune checkpoint inhibitor" OR "immune checkpoint inhibitors" OR "tremelimumab" OR "durvalumab" OR "anti-PD-1 antibodies" OR "avelumab" OR "pidilizumab" OR "anti-CTLA-4" OR "anti-PD-1" OR "anti-PD-L1" OR "anti-CTLA4 antibodies" OR "Anti-CTLA4 agents" OR "anti-PD1 agents" OR "anti-PD-L1 agents" OR "CTLA-4–blocking antibody" OR "lambrolizumab") ANDTOPIC: (“cutaneous side effects” OR “cutaneous adverse reactions” OR “skin toxicity” OR “skin toxicity events” OR “skin manifestations” OR “cutaneous reactions” OR “cutaneous reaction” OR “Manifestation Skin” OR “Manifestations Skin” OR “Skin Manifestation” OR “Skin Manifestations” OR “Drug eruptions” OR “Drug Eruption” OR “Eruption Drug” OR “dermatological reaction” OR “dermatological reactions” OR “skin-related toxicities” OR “cutaneous adverse effects” OR “cutaneous adverse effect” OR “dermatologic toxicities” OR “dermatologic toxicity” OR "rash" OR "skin rash" OR "skin rashes" OR "pruritus" OR "vitiligo" OR "drug reaction" OR "macular eruption" OR "papular eruption" OR "maculopapular eruption" OR "skin eruption" OR "erythema" OR "desquamation" OR "erythroderma" OR "vesicular eruption" OR "exfoliative dermatitis" OR "ulcerative dermatitis" OR "bullous dermatitis" OR "dermatitis" OR "eczema" OR "skin-related toxicity" OR "lichenoid dermatitis" OR "xerosis" OR "alopecia" OR "urticaria" OR "photosensitivity reaction" OR "hyperhidrosis" OR "skin exfoliation" OR "hair color changes" OR "maculopapular exanthema" OR "rosacea" OR "pyoderma gangrenosum" OR "acneiform rash" OR "cutaneous sarcoidosis" OR "dermatomyositis" OR "DRESS syndrome" OR "Stevens Johnson syndrome" OR "toxic epidermal necrolysis" OR "toxic epidermal necrosis" OR "psoriasiform eruption" OR "mucositis" OR "hand-foot syndrome" OR "hypopigmentation" OR "sweet syndrome" OR "Grover disease" OR "hair depigmentation" OR "psoriasis" OR "bullous pemphigoid" OR "alopecia areata" OR "lichenoid reactions" OR "vasculitis" OR "cellulitis" OR "sclerodermoid reaction" OR "cutaneous adverse effects" OR "actinic keratoses" OR "squamous cell carcinoma" OR "seborrheic keratoses" OR "lichen planus" OR "hair growth" OR "hyperkeratosis" OR "atopic dermatitis" OR "stomatitis" OR "dermatitis herpetiformis" OR "oral lichenoid reaction" OR "Dysgeusia" OR "ulcerations" OR "rash maculopapular" OR "erythematous rash" OR "pruritic rash" OR "drug eruption" OR "papules" OR "plaques" OR "vitiligo-like" OR "depigmentation" OR "pruritic maculopapular rash")  Índices: SCI-EXPANDED, SSCI, A&HCI, CPCI-S, CPCI-SSH, ESCI. Stipulated time: Every year |  |

**GOOGLE Scholar**

| **Search Strategy** | **Results** |
| --- | --- |
| Cancer, checkpoint inhibitor, Skin Manifestations, skin-related toxicities | 324 |

**OPEN GRAY**

| **Search Strategy** | **Results** |
| --- | --- |
| Cancer, checkpoint inhibitor | 13 |
