# Supplementary material for: Prevalence of dermatological toxicities in patients with melanoma undergoing immunotherapy: Systematic review and meta-analysis
Source: PLoS One. 2021 Aug 6;16(8):e0255716. doi: 10.1371/journal.pone.0255716 (PMC8345892; doi:10.1371/journal.pone.0255716)
Supplement: S2 File — (DOCX) [file pone.0255716.s005.docx]

S2 File– Articles excluded after full text reading, phase 2 (n=184).

Continue...

| **Author, Year** | **Reason of exclusion** |
| --- | --- |
| Ahmad et al, 2015 | 5 |
| Alley et al, 2017 | 10 |
| Antonia, 2017 | 3 |
| Areses Manrique, 2018 | 10 |
| Atkins et al, 2018 | 4 |
| Bahig et al, 2019 | 6 |
| Balar et al, 2017 | 10 |
| Bang, et al, 2017 | 8 |
| Bauml et al, 2017 | 10 |
| Beer et al, 2017 | 10 |
| Bordoni et al, 2018 | 5 |
| Brahmer et al, 2015 | 10 |
| Brahmer et al, 2012 | 8 |
| Calabrò et al, 2013 | 10 |
| Calvo et al, 2015 | 3 |
| Campian et al, 2017 | 3 |
| Carbone et al, 2017 | 3 |
| Chang et al, 2018 | 4 |
| Chen et al, 2017 | 10 |
| Chesney et al, 2018 | 4 |
| Cheung et al, 2019 | 7 |
| Chiarion Sileni et al, 2014 | 5 |
| Chow et al, 2016 | 3 |
| Cohen et al, 2019 | 2 |
| Cohen et al, 2018 | 5 |
| Coleman et al, 2019 | 8 |
| D`angelo et al, 2018 | 10 |
| De Giorgi et al, 2019 | 10 |
| Del Vecchio et al, 2014 | 5 |
| Di Giacomo et al, 2015 | 4 |
| Doi et al, 2018 | 10 |
| Duffy et al, 2017 | 4 |
| Fang et al, 2018 | 10 |
| Fehrenbacher et al, 2016 | 6 |
| Fehrenbacher et al, 2018 | 5 |
| Feng et al, 2017 | 5 |
| Fennell et al, 2018 | 3 |
| Ferris et al, 2016 | 10 |
| Ferris et al, 2018 | 5 |
| Finkelmeier et al, 2019 | 10 |
| Freeman-Keller et al, 2016 | 5 |
| Frenel et al, 2017 | 10 |
| Fuchs et al, 2018 | 10 |
| Fujii et al, 2018 | 8 |
| Gadgeel et al, 2018 | 10 |
| Garassino et al, 2018 | 10 |
| Garon et al, 2015 | 10 |
| Gettinger et al, 2016 | 10 |
| Gettinger et al, 2015 | 10 |
| Gibney et al, 2015 | 1 |
| Goldinger et al, 2016 | 5 |
| Govidan et al, 2017 | 10 |
| Groisberg et al, 2017 | 8 |
| Grossi et al, 2018 | 10 |
| Gulley et al, 2017 | 6  Continue... |
| Hamid et al, 2017 | 5 |
| Haratani et al, 2018 | 10 |
| Hasan Ali et al, 2016 | 10 |
| Hasan Ali et al, 2019 | 10 |
| Hellmann et al, 2017 | 10 |
| Heppt et al, 2016 | 4 |
| Herbst et al, 2016 | 10 |
| Hida et al, 2017 | 10 |
| Hofmann et al, 2016 | 8 |
| Hsu et al, 2017 | 10 |
| Hsu et al, 2018 | 8 |
| Huang et al, 2019 | 10 |
| Hur et al, 2019 | 10 |
| Hwang et al, 2016 | 3 |
| Hwang et al, 2016 | 8 |
| Indini et al, 2019 | 8 |
| Iyer et al, 2018 | 4 |
| Jamal et al, 2014 | 3 |
| Janjigian et al, 2018 | 10 |
| Johnson et al, 2015 | 6 |
| Johnson et al, 2015 | 8 |
| Kanda et al, 2016 | 10 |
| Kang et al, 2017 | 10 |
| Khouri et al, 2018 | 4 |
| Kim et al, 2013 | 2 |
| King-Kallimanis et al, 2018 | 10 |
| Kiyota et al, 2017 | 5 |
| Knispel et al, 2018 | 2  Continue... |
| Komatsu et al, 2018 | 6 |
| Koshkin et al, 2018 | 10 |
| Kudo et al, 2017 | 10 |
| Larkin et al, 2015 | 5 |
| Lee et al, 2018 | 10 |
| Lesokhin et al, 2016 | 10 |
| Levy et al, 2016 | 6 |
| Liniker et al, 2016 | 8 |
| Lisberg et al, 2018 | 5 |
| Lomax et al, 2016 | 10 |
| Luke et al, 2015 | 4 |
| Madan et al, 2012 | 1 |
| Maio et al, 2015 | 5 |
| Maio et al, 2017 | 10 |
| Maio et al, 2018 | 2 |
| Malero et al, 2016 | 3 |
| Maruyama et al, 2017 | 10 |
| McArthur et al, 2016 | 4 |
| McDermott et al, 2013 | 1 |
| McNeel et al, 2012 | 4 |
| Mehra et al, 2018 | 5 |
| Menzies et al, 2017 | 9 |
| Migden et al, 2018 | 10 |
| Min Lee et al, 2018 | 10 |
| Mitchell et al, 2018 | 4 |
| Mo et al, 2018 | 10 |
| Morris et al, 2017 | 10 |
| Motzer et al, 2015 | 10  Continue... |
| Motzer et al, 2015 | 3 |
| Naqash et al, 2019 | 2 |
| Ott et al, 2017 | 10 |
| Ott et al, 2017 | 6 |
| Overman et al, 2017 | 10 |
| Papadimitrakopoulou et al, 2015 | 3 |
| Plimack et al, 2017 | 10 |
| Powles et al, 2018 | 10 |
| Puzanov et al, 2016 | 1 |
| Reck et al, 2013 | 10 |
| Reck et al, 2016 | 10 |
| Reck et al, 2016 | 10 |
| Reilley et al, 2017 | 4 |
| Riaz et al, 2018 | 3 |
| Ribas et al, 2005 | 8 |
| Ribas et al, 2016 | 5 |
| Rittmeyer et al, 2017 | 6 |
| Rizvi et al, 2014 | 3 |
| Rizvi et al, 2015 | 10 |
| Rizvi et al, 2016 | 10 |
| Robert et al, 2013 | 1 |
| Robert et al, 2014 | 5 |
| Rosenberg et al, 2016 | 10 |
| Sakamuri et al, 2018 | 4 |
| Sampson et al, 2015 | 3 |
| Sanlorenzo et al, 2015 | 5 |
| Sato et al, 2018; | 10 |
| Schiavone et al, 2016 | 3  Continue... |
| Scholz et al, 2017 | 1 |
| Seiwert et al, 2016 | 10 |
| Shah et al, 2018 | 10 |
| Sharma et al, 2016 | 5 |
| Sharma et al, 2017 | 10 |
| Shen et al, 2018 | 5 |
| Shi et al, 2016 | 8 |
| Shimizu et al, 2016; | 10 |
| Siegel et al, 2018 | 8 |
| Siu et al, 2018 | 10 |
| Slovin et al, 2013 | 10 |
| Small et al, 2007 | 10 |
| Strauss et al, 2018 | 4 |
| Sun et al, 2018 | 4 |
| Tahara et al, 2018 | 5 |
| Tarhini et al, 2012 | 4 |
| Tarhini et al, 2014 | 4 |
| Taylor et al, 2015 | 3 |
| Teraoka et al, 2017 | 10 |
| Theurich et al, 2016 | 6 |
| Thronicke et al, 2017 | 4 |
| Toi et al, 2017 | 10 |
| Tomita et al, 2017 | 4 |
| Toon et al, 2014 | 3 |
| Topalian et al, 2012 | 8 |
| Tournoy et al, 2018 | 6 |
| Varga et al. 2018 | 10 |
| Vokes et al, 2018 | 10  Continue... |
| Von Reibnitz et al, 2018 | 8 |
| Vonderheide et al, 2010 | 4 |
| Wang et al, 2018 | 8 |
| Weber et al, 2017 | 5 |
| Weiss et al, 2017 | 10 |
| Welborn et al, 2018 | 4 |
| Wilgenhof et al, 2016 | 4 |
| Wolchok et al, 2011 | 3 |
| Wolchok et al, 2017 | 5 |
| Yamamoto et al, 2016 | 10 |
| Yamazaki et al, 2015 | 8 |
| Yang et al, 2019 | 2 |
| Younes et al, 2016 | 10 |
| Younes et al, 2019 | 2 |
| Zandberg et al, 2018 | 10 |
| Zhang et al, 2018 | 2 |
| Zhu et al, 2018 | 2 |

Source: Elaborated by the authors

Continuation...

1- Immune checkpoint study associated with a vaccine (n=6); 2- Immune checkpoint study with target therapy (n=9); 3- Reviews of the literature, letters, case reports, protocols (n=18); 4- Immune checkpoint study associated with other therapies (n=23); 5- Complementary studies with duplicate data (n=24); 6- Absence of dermatological toxicity (n=10); 7- Qualitative study (n=1); 8- Data extraction not possible/ Incomplete (n=18); 9- Pre-existing autoimmune disease (n=1); 10- Other types of cancer (not melanoma) (n=74).

**REFERENCES**

1. Ahmad SS, Qian W, Ellis S, Mason E, Khattak MA, Gupta A et al. Ipilimumab in the real world: the UK expanded access programme experience in previously treated advanced melanoma patients. Melanoma Res. 2015 Oct; 25 (5): 432-42.

2. Alley EW, Lopez J, Santoro A, Morosky A, Saraf S, Piperdi B et al. Clinical safety and activity of pembrolizumab in patients with malignant pleural mesothelioma (KEYNOTE-028): preliminary results from a non-randomised, open-label, phase 1b trial. Lancet Oncol. 2017 May; 18 (5): 623-630.

3. Antonia SJ. Durvalumab after Chemoradiotherapy in Stage III Non-Small-Cell Lung Cancer. Reply. N Engl J Med. 2019 Mar 7; 380 (10): 990.

4. Areses Manrique MC, Mosquera Martínez J, García González J, Afonso Afonso FJ, Lázaro Quintela M, Fernández Núñez N et al. Real world data of nivolumab for previously treated non-small cell lung cancer patients: a Galician lung cancer group clinical experience. Transl Lung Cancer Res. 2018 Jun; 7 (3): 404-415.

5. Atkins MB, Hodi FS, Thompson JA, McDermott DF, Hwu WJ, Lawrence DP et al. Pembrolizumab Plus Pegylated Interferon alfa-2b or Ipilimumab for Advanced Melanoma or Renal Cell Carcinoma: Dose-Finding Results from the Phase Ib KEYNOTE-029 Study. Clin Cancer Res. 2018 Apr 15; 24 (8): 1805-1815.

6. Bahig H, Aubin F, Stagg J, Gologan O, Ballivy O, Bissada E et al. Phase I/II trial of Durvalumab plus Tremelimumab and stereotactic body radiotherapy for metastatic head and neck carcinoma. BMC Cancer. 2019 Jan 14; 19 (1): 68.

7. Balar AV, Galsky MD, Rosenberg JE, Powles T, Petrylak DP, Bellmunt J et al. Atezolizumab as first-line treatment in cisplatin-ineligible patients with locally advanced and metastatic urotelial carcinoma: a single-arm, multicentre, phase 2 trial. Lancet. 2017 Jan 7; 389 (10064): 67-76.

8. Bang A, Wilhite TJ, Pike LRG, Cagney DN, Aizer AA, Taylor A et al. Multicenter Evaluation of the Tolerability of Combined Treatment With PD-1 and CTLA-4 Immune Checkpoint Inhibitors and Palliative Radiation Therapy. Int J Radiat Oncol Biol Phys. 2017 Jun 1; 98 (2): 344-351.

9. Bauml J, Seiwert TY, Pfister DG, Worden F, Liu SV, Gilbert J et al. Pembrolizumab for Platinum- and Cetuximab-Refractory Head and Neck Cancer: Results From a Single-Arm, Phase II Study. J Clin Oncol. 2017 May 10; 35 (14): 1542-1549.

10. Beer TM, Kwon ED, Drake CG, Fizazi K, Logothetis C, Gravis G et al. Randomized, Double-Blind, Phase III Trial of Ipilimumab Versus Placebo in Asymptomatic or Minimally Symptomatic Patients With Metastatic Chemotherapy-Naive Castration-Resistant Prostate Cancer. J Clin Oncol. 2017 Jan; 35 (1): 40-47.

11. Bordoni R, Ciardiello F, von Pawel J, Cortinovis D, Karagiannis T, Ballinger M et al. Patient-Reported Outcomes in OAK: A Phase III Study of Atezolizumab Versus Docetaxel in Advanced Non-Small-cell Lung Cancer. Clin Lung Cancer. 2018 Sep; 19 (5): 441-449.

12. Brahmer J, Reckamp KL, Baas P, Crinò L, Eberhardt WE, Poddubskaya E et al. Nivolumab versus Docetaxel in Advanced Squamous-Cell Non-Small-Cell Lung Cancer. N Engl J Med. 2015 Jul 9; 373 (2): 123-35.

13. Brahmer JR, Tykodi SS, Chow LQ, Hwu WJ, Topalian SL, Hwu P et al. Safety and activity of anti-PD-L1 antibody in patients with advanced cancer. N Engl J Med. 2012 Jun 28; 366 (26): 2455-65.

14. Calabrò L, Morra A, Fonsatti E, Cutaia O, Amato G, Giannarelli D et al. Tremelimumab for patients with chemotherapy-resistant advanced malignant mesothelioma: an open-label, single-arm, phase 2 trial. Lancet Oncol. 2013 Oct; 14 (11): 1104-1111.

15. Calvo E, López-Martin JA, Bendell J, Eder J, Taylor M, Ott PA et al. Nivolumab (NIVO) monotherapy or in combination with ipilimumab (IPI) for treatment of recurrent small cell lung cancer (SCLC). European Journal of Cancer. 2015 Set; 51 (3): S633.

16. Campian J, Ghiaseddin A, Rahman M, Ansstas G, Kim A, Leuthardt E et al. Early results of a multicenter phase I and open-label, randomized phase ii study testing the toxicities and efficacy of MK-3475 (pembrolizumab) in combination with MRI-guided laser interstitial thermal therapy (LITT) in recurrent malignant gliomas. Neuro Oncol. 2017 Nov; 19 (Suppl 6): vi29-vi30.

17. Carbone DP, Reck M, Paz-Ares L, Creelan B, Horn L, Steins M et al. First-Line Nivolumab in Stage IV or Recurrent Non-Small-Cell Lung Cancer. N Engl J Med. 2017 Jun 22; 376 (25): 2415-2426.

18. Chang EWY, Tai DWM, Koo SL, Matthew CHNG, Yeong JPS, Zhai WW et al. A phase II open-label, single-centre, non-randomized trial of Y90 transarterial radioembolization in combination with nivolumab in Asian patients with intermediate stage hepatocellular carcinoma: An immunological study of radioembolization in combination with anti-PD1 therapy in HCC. Journal of Clinical Oncology. 2018 Sept; 4_suppl, TPS542-TPS542.

19. Chen WC, Chu PY, Lee YT, Lu WB, Liu CY, Chang PM et al. Pembrolizumab for recurrent/metastatic head and neck squamous cell carcinoma in an Asian population. Medicine (Baltimore). 2017 Dec; 96 (52): e9519.

20. Chesney J, Puzanov I, Collichio F, Singh P, Milhem MM, Glaspy J et al. Randomized, Open-Label Phase II Study Evaluating the Efficacy and Safety of Talimogene Laherparepvec in Combination With Ipilimumab Versus Ipilimumab Alone in Patients With Advanced, Unresectable Melanoma. J Clin Oncol. 2018 Jun 10; 36 (17): 1658-1667.

21. Cheung WY, White MK, Bayliss MS, Stroupe A, Lovley A, King-Kallimanis BL et al. Patient-reported treatment-related symptom burden for patients with advanced melanoma in Canada. Support Care Cancer. 2019 Jan; 27 (1): 219-227.

22. Chiarion Sileni V, Pigozzo J, Ascierto PA, Grimaldi AM, Maio M, Di Guardo L et al. Efficacy and safety of ipilimumab in elderly patients with pretreated advanced melanoma treated at Italian centres through the expanded access programme. J Exp Clin Cancer Res. 2014 Apr 4; 33: 30.

23. Chow LQM, Haddad R, Gupta S, Mahipal A, Mehra R, Tahara M et al. Antitumor Activity of Pembrolizumab in Biomarker-Unselected Patients With Recurrent and/or Metastatic Head and Neck Squamous Cell Carcinoma: Results From the Phase Ib KEYNOTE-012 Expansion Cohort. J Clin Oncol. 2016 Nov 10; 34 (32): 3838-3845.

24. Cohen EEW, Soulières D, Le Tourneau C, Dinis J, Licitra L, Ahn MJ et al. Pembrolizumab versus methotrexate, docetaxel, or cetuximab for recurrent or metastatic head-and-neck squamous cell carcinoma (KEYNOTE-040): a randomised, open-label, phase 3 study. Lancet. 2019 Jan 12; 393 (10167): 156-167.

25. Cohen RB, Delord JP, Doi T, Piha-Paul SA, Liu SV, Gilbert J et al. Pembrolizumab for the Treatment of Advanced Salivary Gland Carcinoma: Findings of the Phase 1b KEYNOTE-028 Study. Am J Clin Oncol. 2018 Feb 21. doi: 10.1097/COC.0000000000000429.

26. Coleman E, Ko C, Dai F, Tomayko MM, Kluger H, Leventhal JS. Inflammatory eruptions associated with immune checkpoint inhibitor therapy: A single-institution retrospective analysis with stratification of reactions by toxicity and implications for management. J Am Acad Dermatol. 2019 Apr; 80 (4): 990-997.

27. D'Angelo SP, Mahoney MR, Van Tine BA, Atkins J, Milhem MM, Jahagirdar BN et al. Nivolumab with or without ipilimumab treatment for metastatic sarcoma (Alliance A091401): two open-label, non-comparative, randomised, phase 2 trials. Lancet Oncol. 2018 Mar; 19 (3): 416-426.

28. De Giorgi U, Cartenì G, Giannarelli D, Basso U, Galli L, Cortesi E et al. Safety and efficacy of nivolumab for metastatic renal cell carcinoma: real-world results from na expanded access programme. BJU Int. 2019 Jan; 123 (1): 98-105.

29. Del Vecchio M, Di Guardo L, Ascierto PA, Grimaldi AM, Sileni VC, Pigozzo J,Ferraresi V et al. Efficacy and safety of ipilimumab 3mg/kg in patients with pretreated, metastatic, mucosal melanoma. Eur J Cancer. 2014 Jan; 50 (1): 121-7.

30. Di Giacomo AM, Ascierto PA, Queirolo P, Pilla L, Ridolfi R, Santinami M et al. Three-year follow-up of advanced melanoma patients who received ipilimumab plus fotemustine in the Italian Network for Tumor Biotherapy (NIBIT)-M1 phase II study. Ann Oncol. 2015 Apr; 26 (4): 798-803.

31. Doi T, Piha-Paul SA, Jalal SI, Saraf S, Lunceford J, Koshiji M et al. Safety and Antitumor Activity of the Anti-Programmed Death-1 Antibody Pembrolizumab in Patients With Advanced Esophageal Carcinoma. J Clin Oncol. 2018 Jan; 36 (1): 61-67.

32. Duffy AG, Ulahannan SV, Makorova-Rusher O, Rahma O, Wedemeyer H, Pratt D et al. Tremelimumab in combination with ablation in patients with advanced hepatocellular carcinoma. J Hepatol. 2017 Mar; 66 (3): 545-551.

33. Fang W, Yang Y, Ma Y, Hong S, Lin L, He X et al. Camrelizumab (SHR-1210) alone or in combination with gemcitabine plus cisplatin for nasopharyngeal carcinoma: results from two single-arm, phase 1 trials. Lancet Oncol. 2018 Oct; 19 (10): 1338-1350.

34. Fehrenbacher L, Spira A, Ballinger M, Kowanetz M, Vansteenkiste J, Mazieres J et al. Atezolizumab versus docetaxel for patients with previously treated non-small-cell lung cancer(POPLAR): a multicentre, open-label, phase 2 randomised controlled trial. Lancet. 2016 Apr 30; 387 (10030): 1837-46.

35. Fehrenbacher L, von Pawel J, Park K, Rittmeyer A, Gandara DR, Ponce Aix S et al. Updated Efficacy Analysis Including Secondary Population Results for OAK: A Randomized Phase III Study of Atezolizumab versus Docetaxel in Patients with Previously Treated Advanced Non-Small Cell Lung Cancer. J Thorac Oncol. 2018 Aug; 13 (8): 1156-1170.

36. Feng Y, Wang X, Bajaj G, Agrawal S, Bello A, Lestini B et al. Nivolumab Exposure-Response Analyses of Efficacy and Safety in Previously Treated Squamous or Nonsquamous Non-Small Cell Lung Cancer. Clin Cancer Res. 2017 Sep 15; 23 (18): 5394-5405.

37. Fennell DA. Programmed Death 1 Blockade With Nivolumab in Patients With Recurrent Malignant Pleural Mesothelioma. J Thorac Oncol. 2018 Oct; 13 (10): 1436-1437.

38. Ferris RL, Blumenschein G Jr, Fayette J, Guigay J, Colevas AD, Licitra L et al. Nivolumab for Recurrent Squamous-Cell Carcinoma of the Head and Neck. N Engl J Med. 2016 Nov 10; 375 (19): 1856-1867.

39. Ferris RL, Blumenschein G Jr, Fayette J, Guigay J, Colevas AD, Licitra L et al. Nivolumab vs investigator's choice in recurrent or metastatic squamous cell carcinoma of the head and neck: 2-year long-term survival update of CheckMate 141 with analyses by tumor PD-L1 expression. Oral Oncol. 2018 Jun; 81: 45-51.

40. Finkelmeier F, Czauderna C, Perkhofer L, Ettrich TJ, Trojan J, Weinmann A et al. Feasibility and safety of nivolumab in advanced hepatocellular carcinoma: real-life experience from three German centers. J Cancer Res Clin Oncol. 2019 Jan;145 (1): 253-259.

41. Freeman-Keller M, Kim Y, Cronin H, Richards A, Gibney G, Weber JS. Nivolumab in Resected and Unresectable Metastatic Melanoma: Characteristics of Immune-Related Adverse Events and Association with Outcomes. Clin Cancer Res. 2016 Feb 15; 22 (4): 886-94.

42. Frenel JS, Le Tourneau C, O'Neil B, Ott PA, Piha-Paul SA, Gomez-Roca C et al. Safety and Efficacy of Pembrolizumab in Advanced, Programmed Death Ligand 1-Positive Cervical Cancer: Results From the Phase Ib KEYNOTE-028 Trial. J Clin Oncol. 2017 Dec 20; 35 (36): 4035-4041.

43. Fuchs CS, Doi T, Jang RW, Muro K, Satoh T, Machado M et al. Safety and Efficacy of Pembrolizumab Monotherapy in Patients With Previously Treated Advanced Gastric and Gastroesophageal Junction Cancer: Phase 2 Clinical KEYNOTE-059 Trial. JAMA Oncol. 2018 May 10; 4 (5): e180013.

44. Fujii T, Colen RR, Bilen MA, Hess KR, Hajjar J, Suarez-Almazor ME et al. Incidence of immune-related adverse events and its association with treatment outcomes: the MD Anderson Cancer Center experience. Invest New Drugs. 2018 Aug; 36 (4): 638-646.

45. Gadgeel SM, Stevenson JP, Langer CJ, Gandhi L, Borghaei H, Patnaik A et al. Pembrolizumab and platinum-based chemotherapy as first-line therapy for advanced non-small-cell lung cancer: Phase 1 cohorts from the KEYNOTE-021 study. Lung Cancer. 2018 Nov; 125: 273-281.

46. Garassino MC, Crinò L, Catino A, Ardizzoni A, Cortesi E, Cappuzzo F et al. Nivolumab in never-smokers with advanced squamous non-small cell lung cancer: Results from the Italian cohort of an expanded access program. Tumour Biol. 2018 Nov; 40 (11): 1010428318815047.

47. Garon EB, Rizvi NA, Hui R, Leighl N, Balmanoukian AS, Eder JP et al. Pembrolizumab for the treatment of non-small-cell lung cancer. N Engl J Med. 2015 May 21; 372 (21): 2018-28.

48. Gettinger S, Rizvi NA, Chow LQ, Borghaei H, Brahmer J, Ready N et al. Nivolumab Monotherapy for First-Line Treatment of Advanced Non-Small-Cell Lung Cancer. J Clin Oncol. 2016 Sep 1; 34 (25): 2980-7.

49. Gettinger SN, Horn L, Gandhi L, Spigel DR, Antonia SJ, Rizvi NA et al. Overall Survival and Long-Term Safety of Nivolumab (Anti-Programmed Death 1 Antibody, BMS-936558, ONO-4538) in Patients With Previously Treated Advanced Non-Small-Cell Lung Cancer. J Clin Oncol. 2015 Jun 20; 33 (18): 2004-12.

50. Gibney GT, Kudchadkar RR, DeConti RC, Thebeau MS, Czupryn MP, Tetteh L et al. Safety, correlative markers, and clinical results of adjuvant nivolumab in combination with vaccine in resected high-risk metastatic melanoma. Clin Cancer Res. 2015 Feb 15; 21 (4): 712-20.

51. Goldinger SM, Stieger P, Meier B, Micaletto S, Contassot E, French LE et al. Cytotoxic Cutaneous Adverse Drug Reactions during Anti-PD-1 Therapy. Clin Cancer Res. 2016 Aug 15; 22 (16): 4023-9.

52. Govindan R, Szczesna A, Ahn MJ, Schneider CP, Gonzalez Mella PF, Barlesi F et al. Phase III Trial of Ipilimumab Combined With Paclitaxel and Carboplatin in Advanced Squamous Non-Small-Cell Lung Cancer. J Clin Oncol. 2017 Oct 20; 35 (30): 3449-3457.

53. Groisberg R, Hong DS, Behrang A, Hess K, Janku F, Piha-Paul S et al. Characteristics and outcomes of patients with advanced sarcoma enrolled in early phase immunotherapy trials. J Immunother Cancer. 2017 Dec 19; 5 (1): 100.

54. Grossi F, Crinò L, Logroscino A, Canova S, Delmonte A, Melotti B et al. Use of nivolumab in elderly patients with advanced squamous non-small-cell lung cancer: results from the Italian cohort of an expanded access programme. Eur J Cancer. 2018 Sep; 100: 126-134.

55. Gulley JL, Rajan A, Spigel DR, Iannotti N, Chandler J, Wong DJL et al. Avelumab for patients with previously treated metastatic or recurrent non-small-cell lung cancer (JAVELIN Solid Tumor): dose-expansion cohort of a multicentre, open-label, phase 1b trial. Lancet Oncol. 2017 May; 18 (5): 599-610.

56. Hamid O, Puzanov I, Dummer R, Schachter J, Daud A, Schadendorf D et al. Final analysis of a randomised trial comparing pembrolizumab versus investigator-choice chemotherapy for ipilimumab-refractory advanced melanoma. Eur J Cancer. 2017. Nov; 86: 37-45.

57. Haratani K, Hayashi H, Chiba Y, Kudo K, Yonesaka K, Kato R et al. Association of Immune-Related Adverse Events With Nivolumab Efficacy in Non-Small-Cell Lung Cancer. JAMA Oncol. 2018 Mar 1; 4 (3): 374-378.

58. Hasan Ali O, Berner F, Bomze D, Fässler M, Diem S, Cozzio A, Jörger M, Früh M, Driessen C, Lenz TL, Flatz L. Human leukocyte antigen variation is associated with adverse events of checkpoint inhibitors. Eur J Cancer. 2019 Jan; 107: 8-14.

59. Hasan Ali O, Diem S, Markert E, Jochum W, Kerl K, French LE et al. Characterization of nivolumab-associated skin reactions in patients with metastatic non-small cell lung cancer. Oncoimmunology. 2016 Sep 19; 5 (11): e1231292.

60. Hellmann MD, Rizvi NA, Goldman JW, Gettinger SN, Borghaei H, Brahmer JR et al. Nivolumab plus ipilimumab as first-line treatment for advanced non-small-cell lung cancer (CheckMate 012): results of an open-label,phase 1, multicohort study. Lancet Oncol. 2017 Jan; 18 (1): 31-41.

61. Heppt MV, Eigentler TK, Kähler KC, Herbst RA, Göppner D, Gambichler T et al. Immune checkpoint blockade with concurrent electrochemotherapy in advanced melanoma: a retrospective multicenter analysis. Cancer Immunol Immunother. 2016 Aug; 65 (8): 951-9.

62. Herbst RS, Baas P, Kim DW, Felip E, Pérez-Gracia JL, Han JY et al. Pembrolizumab versus docetaxel for previously treated, PD-L1-positive, advanced non-small-cell lung cancer (KEYNOTE-010): a randomised controlled trial. Lancet. 2016 Apr 9; 387 (10027): 1540-50.

63. Hida T, Nishio M, Nogami N, Ohe Y, Nokihara H, Sakai H et al. Efficacy and safety of nivolumab in Japanese patients with advanced or recurrent squamous non-small cell lung cancer. Cancer Sci. 2017 May; 108 (5): 1000-1006.

64. Hofmann L, Forschner A, Loquai C, Goldinger SM, Zimmer L, Ugurel S et al. Cutaneous, gastrointestinal, hepatic, endocrine, and renal side-effects of anti-PD-1 therapy. Eur J Cancer. 2016 Jun; 60: 190-209.

65. Hsu C, Lee SH, Ejadi S, Even C, Cohen RB, Le Tourneau C et al. Safety and Antitumor Activity of Pembrolizumab in Patients With Programmed Death-Ligand 1-Positive Nasopharyngeal Carcinoma: Results of the KEYNOTE-028 Study. J Clin Oncol. 2017 Dec 20; 35 (36): 4050-4056.

66. Hsu JC, Lin JY, Hsu MY, Lin PC. Effectiveness and safety of immune checkpoint inhibitors: A retrospective study in Taiwan. PLoS One. 2018 Aug 24; 13 (8): e0202725.

67. Huang J, Mo H, Zhang W, Chen X, Qu D, Wang X et al. Promising efficacy of SHR-1210, a novel anti-programmed cell death 1 antibody, in patients with advanced gastric and gastroesophageal junction cancer in China. Cancer. 2019 Mar 1; 125 (5): 742-749.

68. Hur JY, Kim Y, Kwon GY, Kang M, Sung HH, Jeon HG et ak. Atezolizumab in Patients with Pretreated Urothelial Cancer: a Korean Single-Center, Retrospective Study. Cancer Res Treat. 2019 Jan 9. doi: 10.4143/crt.2018.604.

69. Hwang SJ, Carlos G, Chou S, Wakade D, Carlino MS, Fernandez-Penas P. Bullous pemphigoid, an autoantibody-mediated disease, is a novel immune-related adverse event in patients treated with anti-programmed cell death 1 antibodies. Melanoma Res. 2016 Aug; 26 (4): 413-6.

70. Hwang SJ, Carlos G, Wakade D, Byth K, Kong BY, Chou S et al. Cutaneous adverse events (AEs) of anti-programmed cell death(PD)-1 therapy in patients with metastatic melanoma: A single-institution cohort. J Am Acad Dermatol. 2016 Mar; 74 (3): 455-61. e1.

71. Indini A, Di Guardo L, Cimminiello C, Prisciandaro M, Randon G, De Braud F et al. Immune-related adverse events correlate with improved survival in patients undergoing anti-PD1 immunotherapy for metastatic melanoma. J Cancer Res Clin Oncol. 2019 Feb; 145 (2): 511-521.

72. Iyer PC, Dadu R, Gule-Monroe M, Busaidy NL, Ferrarotto R, Habra MA et al. Salvage pembrolizumab added to kinase inhibitor therapy for the treatment of anaplastic thyroid carcinoma. J Immunother Cancer. 2018 Jul 11; 6 (1): 68.

73. Jamal R, Belanger K, Friedmann JE, Ayoub JPM, Cocolakis E, Kazemi S et al. A randomized phase II study of ipilimumab (IPI) with carboplatin and paclitaxel (CP) in patients with unresectable stage III or IV metastatic melanoma (MM). Journal of Clinical Oncology. 2014; 32: 15_suppl, 9066-9066.

74. Janjigian YY, Bendell J, Calvo E, Kim JW, Ascierto PA, Sharma P et al. CheckMate-032 Study: Efficacy and Safety of Nivolumab and Nivolumab Plus Ipilimumab in Patients With Metastatic Esophagogastric Cancer. J Clin Oncol. 2018 Oct 1; 36 (28): 2836-2844.

75. Johnson DB, Friedman DL, Berry E, Decker I, Ye F, Zhao S et al. Survivorship in Immune Therapy: Assessing Chronic Immune Toxicities, Health Outcomes, and Functional Status among Long-term Ipilimumab Survivors at a Single Referral Center. Cancer Immunol Res. 2015 May; 3 (5): 464-9.

76. Johnson DB, Peng C, Abramson RG, Ye F, Zhao S, Wolchok JD et al. Clinical activity of ipilimumab in acral aelanoma: A retrospective review. Oncologist. 2015 Jun; 20 (6): 648-52.

77. Kanda S, Goto K, Shiraishi H, Kubo E, Tanaka A, Utsumi H et al. Safety and efficacy of nivolumab and standard chemotherapy drug combination in patients with advanced non-small-cell lung cancer: a four arms phase Ib study. Ann Oncol. 2016 Dec; 27 (12): 2242-2250.

78. Kang YK, Boku N, Satoh T, Ryu MH, Chao Y, Kato K et al. Nivolumab in patients with advanced gastric or gastro-oesophageal junction cancer refractory to, or intolerant of, at least two previous chemotherapy regimens (ONO-4538-12, ATTRACTION-2): a randomised, double-blind, placebo-controlled, phase 3 trial. Lancet. 2017 Dec 2; 390 (10111): 2461-2471.

79. Khouri IF, Fernandez Curbelo I, Turturro F, Jabbour EJ, Milton DR, Bassett RL Jr et al. Ipilimumab plus Lenalidomide after Allogeneic and Autologous Stem Cell Transplantation for Patients with Lymphoid Malignancies. Clin Cancer Res. 2018 Mar 1; 24 (5): 1011-1018.

80. Kim KB, Kefford R, Pavlick AC, Infante JR, Ribas A, Sosman JA et al. Phase II study of the MEK1/MEK2 inhibitor Trametinib in patients with metastatic BRAF-mutant cutaneous melanoma previously treated with or without a BRAF inhibitor. J Clin Oncol. 2013 Feb 1; 31 (4): 482-9.

81. King-Kallimanis BL, Kanapuru B, Blumenthal GM, Theoret MR, Kluetz PG. Age-related differences in patient-reported outcomes in patients with advanced lung cancer receiving anti-PD-1/PD-L1 therapy. Semin Oncol. 2018 Aug; 45 (4): 201-209.

82. Kiyota N, Hasegawa Y, Takahashi S, Yokota T, Yen CJ, Iwae S et al. A randomized, open-label, Phase III clinical trial of nivolumab vs. therapy of investigator's choice in recurrent squamous cell carcinoma of the head and neck: A subanalysis of Asian patients versus the global population in checkmate 141. Oral Oncol. 2017 Oct; 73: 138-146.

83. Knispel S, Zimmer L, Kanaki T, Ugurel S, Schadendorf D, Livingstone E. The safety and efficacy of dabrafenib and trametinib for the treatment of melanoma. Expert Opin Drug Saf. 2018 Jan; 17 (1): 73-87.

84. Komatsu T, Konishi K, Aoshima M, Tokura Y, Nakamura K.Combined radiotherapy with nivolumab for extracranial metastatic malignant melanoma. Jpn J Radiol. 2018 Dec; 36 (12): 712-718.

85. Koshkin VS, Barata PC, Zhang T, George DJ, Atkins MB, Kelly WJ et al. Clinical activity of nivolumab in patients with non-clear cell renal cell carcinoma. J Immunother Cancer. 2018 Jan 29; 6 (1): 9.

86. Kudo T, Hamamoto Y, Kato K, Ura T, Kojima T, Tsushima T et al. Nivolumab treatment for oesophageal squamous-cell carcinoma: na open-label, multicentre, phase 2 trial. Lancet Oncol. 2017 May;18 (5): 631-639.

87. Larkin J, Lao CD, Urba WJ, McDermott DF, Horak C, Jiang J. Efficacy and Safety of Nivolumab in Patients with BRAF V600 Mutant and BRAF Wild-Type Advanced Melanoma: A Pooled Analysis of 4 Clinical Trials. JAMA Oncol. 2015 Jul; 1 (4): 433-40.

88. Lee JS, Lee KH, Cho EK, Kim DW, Kim SW, Kim JH et al. Nivolumab in advanced non-small-cell lung cancer patients who failed prior platinum-based chemotherapy. Lung Cancer. 2018 Aug; 122: 234-242.

89. Lesokhin AM, Ansell SM, Armand P, Scott EC, Halwani A, Gutierrez M et al. Nivolumab in Patients With Relapsed or Refractory Hematologic Malignancy: Preliminary Results of a Phase Ib Study. J Clin Oncol. 2016 Aug 10; 34 (23): 2698-704.

90. Levy A, Massard C, Soria JC, Deutsch E. Concurrent irradiation with the anti-programmed cell death ligand-1 immune checkpoint blocker durvalumab: Single centre subset analysis from a phase 1/2 trial. Eur J Cancer. 2016 Nov; 68: 156-162.

91. Liniker E, Menzies AM, Kong BY, Cooper A, Ramanujam S, Lo S et al. Activity and safety of radiotherapy with anti-PD-1 drug therapy in patients with metastatic melanoma. Oncoimmunology. 2016 Aug 19; 5 (9): e1214788.

92. Lisberg A, Tucker DA, Goldman JW, Wolf B, Carroll J, Hardy A et al. Treatment-Related Adverse Events Predict Improved Clinical Outcome in NSCLC Patients on KEYNOTE-001 at a Single Center. Cancer Immunol Res. 2018 Jan 30. doi: 10.1158/2326-6066.CIR-17-0063.

93. Lomax AJ, Beith J, Bhadri V, Boyer M, Grimison P, Horvath LG et al. Outcomes of patients with non-melanoma solid tumours receiving self-funded pembrolizumab at Chris O'Brien Lifehouse. Intern Med J. 2016 Dec; 46 (12): 1392-1398.

94. Luke JJ, Donahue H, Nishino M, Giobbie-Hurder A, Davis M, Bailey N et al. Single Institution Experience of Ipilimumab 3 mg/kg with Sargramostim (GM-CSF) in Metastatic Melanoma. Cancer Immunol Res. 2015 Sep; 3 (9): 986-91.

95. Madan RA, Mohebtash M, Arlen PM, Vergati M, Rauckhorst M, Steinberg SM et al. Ipilimumab and a poxviral vaccine targeting prostate-specific antigen in metastatic castration-resistant prostate cancer: a phase 1 dose-escalation trial. Lancet Oncol. 2012 May; 13 (5): 501-8.

96. Maio M, Grob JJ, Aamdal S, Bondarenko I, Robert C, Thomas L et al. Five-year survival rates for treatment-naive patients with advanced melanoma who received ipilimumab plus dacarbazine in a phase III trial. J Clin Oncol. 2015 Apr 1; 33 (10): 1191-6.

97. Maio M, Lewis K, Demidov L, Mandalà M, Bondarenko I, Ascierto PA et al. Adjuvant vemurafenib in resected, BRAF(V600) mutation-positive melanoma (BRIM8): a randomised, double-blind, placebo-controlled, multicentre, phase 3 trial. Lancet Oncol. 2018 Apr; 19 (4): 510-520.

98. Maio M, Scherpereel A, Calabrò L, Aerts J, Cedres Perez S, Bearz A et al. Tremelimumab as second-line or third-line treatment in relapsed malignant mesothelioma (DETERMINE): a multicentre, international, randomised, double-blind, placebo-controlled phase 2b trial. Lancet Oncol. 2017 Sep; 18 (9): 1261-1273.

99. Malero I, Sangro B, Yau T, Hsu C, Kudo M, Crocenzi T et al. Safety and preliminary efficacy of nivolumab (nivo) in patients (pts) with advanced hepatocellular carcinoma (aHCC): interim analysis of the phase 1/2 CheckMate-040 study. 2016 Dec; 27: 6150.

100. Maruyama D, Hatake K, Kinoshita T, Fukuhara N, Choi I, Taniwaki M et al. Multicenter phase II study of nivolumab in Japanese patients with relapsed or refractory classical Hodgkin lymphoma. Cancer Sci. 2017 May; 108 (5): 1007-1012.

101. McArthur HL, Diab A, Page DB, Yuan J, Solomon SB, Sacchini V et al. A Pilot Study of Preoperative Single-Dose Ipilimumab and/or Cryoablation in Women with Early-Stage Breast Cancer with Comprehensive Immune Profiling. Clin Cancer Res. 2016 Dec 1; 22 (23): 5729-5737.

102. McDermott D, Haanen J, Chen TT, Lorigan P, O'Day S; MDX010-20 Investigators. Efficacy and safety of ipilimumab in metastatic melanoma patients surviving more than 2 years following treatment in a phase III trial (MDX010-20). Ann Oncol. 2013 Oct; 24 (10): 2694-8.

103. McNeel DG, Smith HA, Eickhoff JC, Lang JM, Staab MJ, Wilding G et al. Phase I trial of tremelimumab in combination with short-term androgen deprivation in patients with PSA-recurrent prostate cancer. Cancer Immunol Immunother. 2012 Jul; 61 (7): 1137-47.

104. Mehra R, Seiwert TY, Gupta S, Weiss J, Gluck I, Eder JP et al. Efficacy and safety of pembrolizumab in recurrent/metastatic head and neck squamous cell carcinoma: pooled analyses after long-term follow-up in KEYNOTE-012. Br J Cancer. 2018 Jul; 119 (2): 153-159.

105. Menzies AM, Johnson DB, Ramanujam S, Atkinson VG, Wong ANM, Park JJ et al. Anti-PD-1 therapy in patients with advanced melanoma and preexisting autoimmune disorders or major toxicity with ipilimumab. Ann Oncol. 2017 Feb 1; 28 (2): 368-376.

106. Migden MR, Rischin D, Schmults CD, Guminski A, Hauschild A, Lewis KD et al. PD-1 Blockade with Cemiplimab in Advanced Cutaneous Squamous-Cell Carcinoma. N Engl J Med. 2018 Jul 26; 379 (4): 341-351.

107. Min Lee CK, Li S, Tran DC, Zhu GA, Kim J, Kwong BY, Chang ALS. Characterization of dermatitis after PD-1/PD-L1 inhibitor therapy and association with multiple oncologic outcomes: A retrospective case-control study. J Am Acad Dermatol. 2018 Dec; 79 (6): 1047-1052.

108. Mitchell TC, Hamid O, Smith DC, Bauer TM, Wasser JS, Olszanski AJ et al. Epacadostat Plus Pembrolizumab in Patients With Advanced Solid Tumors: Phase I Results From a Multicenter, Open-Label Phase I/II Trial (ECHO-202/KEYNOTE-037). J Clin Oncol. 2018 Sep 28: JCO2018789602. doi: 10.1200/JCO.2018.78.9602.

109. Mo H, Huang J, Xu J, Chen X, Wu D, Qu D et al. Safety, anti-tumour activity, and pharmacokinetics of fixed-dose SHR-1210, an anti-PD-1 antibody in advanced solid tumours: a dose-escalation, phase 1 study. Br J Cancer. 2018 Aug; 119 (5): 538-545.

110. Morris VK, Salem ME, Nimeiri H, Iqbal S, Singh P, Ciombor K et al. Nivolumab for previously treated unresectable metastatic anal cancer (NCI9673): a multicentre, single-arm,phase 2 study. Lancet Oncol. 2017 Apr; 18 (4): 446-453.

111. Motzer RJ, Escudier B, Mcdermott D, George S, Hammers H, Srinivas S et al. CheckMate 025: a randomized, open-label, phase Ill study of nivolumab versus everolimus in advanced renal cell carcinoma (RCC). Bju International. 2015 Dec; 116: 17.

112. Motzer RJ, Rini BI, McDermott DF, Redman BG, Kuzel TM, Harrison MR et al. Nivolumab for Metastatic Renal Cell Carcinoma: Results of a Randomized Phase II Trial. J Clin Oncol. 2015 May 1; 33 (13): 1430-7.

113. Naqash AR, File DM, Ziemer CM, Whang YE, Landman P, Googe PB et al. Cutaneous adverse reactions in B-RAF positive metastatic melanoma following sequential treatment with B-RAF/MEK inhibitors and immune checkpoint blockade or vice versa. A single-institutional case-series. J Immunother Cancer. 2019 Jan 8; 7 (1): 4.

114. Ott PA, Bang YJ, Berton-Rigaud D, Elez E, Pishvaian MJ, Rugo HS et al. Safety and Antitumor Activity of Pembrolizumab in Advanced Programmed Death Ligand 1-Positive Endometrial Cancer: Results From the KEYNOTE-028 Study. J Clin Oncol. 2017 Aug 1; 35 (22): 2535-2541.

115. Ott PA, Piha-Paul SA, Munster P, Pishvaian MJ, van Brummelen EMJ, Cohen R et al. Safety and antitumor activity of the anti-PD-1 antibody pembrolizumab in patients with recurrent carcinoma of the anal canal. Ann Oncol. 2017 May 1; 28 (5): 1036-1041.

116. Overman MJ, McDermott R, Leach JL, Lonardi S, Lenz HJ, Morse MA et al. Nivolumab in patients with metastatic DNA mismatch repair-deficient or microsatellite instability-high colorectal cancer (CheckMate 142): an open-label,multicentre, phase 2 study. Lancet Oncol. 2017 Sep; 18 (9): 1182-1191.

117. Papadimitrakopoulou V, Patnaik A, Borghaei H, Stevenson J, Gandhi L, Yang MAGCH et al. Pembrolizumab (pembro; MK-3475) plus platinum doublet chemotherapy (PDC) as front-line therapy for advanced non-small cell lung cancer (NSCLC): KEYNOTE-021 Cohorts A and C. Journal of Clinical Oncology. 2015; 33 (15_suppl): 8031-8031.

118. Plimack ER, Bellmunt J, Gupta S, Berger R, Chow LQ, Juco J et al. Safety and activity of pembrolizumab in patients with locally advanced or metastatic urothelial cancer (KEYNOTE-012): a non-randomised,open-label, phase 1b study. Lancet Oncol. 2017 Feb; 18 (2): 212-220.

119. Powles T, Durán I, van der Heijden MS, Loriot Y, Vogelzang NJ, De Giorgi U et al. Atezolizumab versus chemotherapy in patients with platinum-treated locally advanced or metastatic urothelial carcinoma (IMvigor211): a multicentre, open-label, phase 3 randomised controlled trial. Lancet. 2018 Feb 24; 391 (10122): 748-757.

120. Puzanov I, Milhem MM, Minor D, Hamid O, Li A, Chen L et al. Talimogene Laherparepvec in Combination With Ipilimumab in Previously Untreated, Unresectable Stage IIIB-IV Melanoma. J Clin Oncol. 2016 Aug 1; 34 (22): 2619-26.

121. Reck M, Bondarenko I, Luft A, Serwatowski P, Barlesi F, Chacko R et al. Ipilimumab in combination with paclitaxel and carboplatin as first-line therapy in extensive-disease-small-cell lung cancer: results from a randomized, double-blind, multicenter phase 2 trial. Ann Oncol. 2013 Jan; 24 (1): 75-83.

122. Reck M, Luft A, Szczesna A, Havel L, Kim SW, Akerley W et al. Phase III Randomized Trial of Ipilimumab Plus Etoposide and Platinum Versus Placebo Plus Etoposide and Platinum in Extensive-Stage Small-Cell Lung Cancer. J Clin Oncol. 2016 Nov 1; 34 (31): 3740-3748.

123. Reck M, Rodríguez-Abreu D, Robinson AG, Hui R, Csőszi T, Fülöp A et al. Pembrolizumab versus Chemotherapy for PD-L1-Positive Non-Small-Cell Lung Cancer. N Engl J Med. 2016 Nov 10; 375 (19): 1823-1833.

124. Reilley MJ, Bailey A, Subbiah V, Janku F, Naing A, Falchook G et al. Phase I clinical trial of combination imatinib and ipilimumab in patients with advanced malignancies. J Immunother Cancer. 2017 Apr 18; 5: 35.

125. Riaz R, Leah L, Dana Z. Pembrolizumab Plus Chemotherapy in Patients With Previously Untreated Metastatic Non–small Cell Lung Cancer Without EGFR or ALK Mutations is Superior to Chemotherapy Alone. Clinical Pulmonary Medicine. 2018 Sept; 25 (5): 194-195.

126. Ribas A, Camacho LH, Lopez-Berestein G, Pavlov D, Bulanhagui CA, Millham R et al. Antitumor activity in melanoma and anti-self responses in a phase I trial with the anti-cytotoxic T lymphocyte-associated antigen 4 monoclonal antibody CP-675,206. J Clin Oncol. 2005 Dec 10; 23 (35): 8968-77.

127. Ribas A, Hamid O, Daud A, Hodi FS, Wolchok JD, Kefford R et al. Association of Pembrolizumab With Tumor Response and Survival Among Patients With Advanced Melanoma. JAMA. 2016 Apr 19; 315 (15): 1600-9.

128. Rittmeyer A, Barlesi F, Waterkamp D, Park K, Ciardiello F, von Pawel J et al. Atezolizumab versus docetaxel in patients with previously treated non-small-cell lung cancer (OAK): a phase 3, open-label, multicentre randomised controlled trial. Lancet. 2017 Jan 21; 389 (10066): 255-265.

129. Rizvi NA, Garon EB, Patnaik A, Gandhi L, Leighl NB, Sarkis A et al. Safety and clinical activity of MK-3475 as initial therapy in patients with advanced non-small cell lung cancer (NSCLC). Journal of Clinical Oncology. 2014 May; 32:15_suppl, 8007-8007.

130. Rizvi NA, Hellmann MD, Brahmer JR, Juergens RA, Borghaei H, Gettinger S et al. Nivolumab in Combination With Platinum-Based Doublet Chemotherapy for First-Line Treatment of Advanced Non-Small-Cell Lung Cancer. J Clin Oncol. 2016 Sep 1; 34 (25): 2969-79.

131. Rizvi NA, Mazières J, Planchard D, Stinchcombe TE, Dy GK, Antonia SJ et al. Activity and safety of nivolumab, an anti-PD-1 immune checkpoint inhibitor, for patients with advanced, refractory squamous non-small-cell lung cancer (CheckMate 063): a phase 2, single-arm trial. Lancet Oncol. 2015 Mar; 16 (3): 257-65.

132. Robert C, Ribas A, Wolchok JD, Hodi FS, Hamid O, Kefford R et al. Anti-programmed-death-receptor-1 treatment with pembrolizumab in ipilimumab-refractory advanced melanoma: a randomised dose-comparison cohort of a phase 1 trial. Lancet. 2014 Sep 20; 384 (9948): 1109-17.

133. Robert C, Schadendorf D, Messina M, Hodi FS, O'Day S; MDX010-20 investigators. Efficacy and safety of retreatment with ipilimumab in patients with pretreated advanced melanoma who progressed after initially achieving disease control. Clin Cancer Res. 2013 Apr 15; 19 (8): 2232-9.

134. Rosenberg JE, Hoffman-Censits J, Powles T, van der Heijden MS, Balar AV, Necchi A et al. Atezolizumab in patients with locally advanced and metastatic urothelial carcinoma who have progressed following treatment with platinum-based chemotherapy: a single-arm, multicentre, phase 2 trial. Lancet. 2016 May 7; 387 (10031): 1909-20.

135. Sakamuri D, Glitza IC, Betancourt Cuellar SL, Subbiah V, Fu S, Tsimberidou AM et al. Phase I Dose-Escalation Study of Anti-CTLA-4 Antibody Ipilimumab and Lenalidomide in Patients with Advanced Cancers. Mol Cancer Ther. 2018 Mar; 17 (3): 671-676.

136. Sampson JH, Vlahovic G, Sahebjam S, Omuro AMP, Baehring JM, Hafler DA et al. Preliminary safety and activity of nivolumab and its combination with ipilimumab in recurrent glioblastoma (GBM): CHECKMATE-143. Journal of Clinical Oncology. 2015; 33:15_suppl, 3010-3010.

137. Sanlorenzo M, Vujic I, Daud A, Algazi A, Gubens M, Luna SA et al. Pembrolizumab Cutaneous Adverse Events and Their Association With Disease Progression. JAMA Dermatol. 2015 Nov; 151 (11): 1206-1212.

138. Sato K, Akamatsu H, Murakami E, Sasaki S, Kanai K, Hayata A et al. Correlation between immune-related adverse events and efficacy in non-small cell lung cancer treated with nivolumab. Lung Cancer. 2018 Jan; 115: 71-74.

139. Schiavone MB, Broach V, Shoushtari AN, Carvajal RD, Alektiar K, Kollmeier MA et al. Combined immunotherapy and radiation for treatment of mucosal melanomas of the lower genital tract. Gynecol Oncol Rep. 2016 Apr 14; 16: 42-6.

140. Scholz M, Yep S, Chancey M, Kelly C, Chau K, Turner J et al. Phase I clinical trial of sipuleucel-T combined with escalating doses of ipilimumab in progressive metastatic castrate-resistant prostate cancer. Immunotargets Ther. 2017 Mar 20; 6: 11-16.

141. Seiwert TY, Burtness B, Mehra R, Weiss J, Berger R, Eder JP et al. Safety and clinical activity of pembrolizumab for treatment of recurrent or metastatic squamous cell carcinoma of the head and neck (KEYNOTE-012): an open-label, multicentre, phase 1b trial. Lancet Oncol. 2016 Jul; 17 (7): 956-965.

142. Shah MA, Kojima T, Hochhauser D, Enzinger P, Raimbourg J, Hollebecque A et al. Efficacy and Safety of Pembrolizumab for Heavily Pretreated Patients With Advanced, Metastatic Adenocarcinoma or Squamous Cell Carcinoma of the Esophagus: The Phase 2 KEYNOTE-180 Study. JAMA Oncol. 2018 Dec 20. doi: 10.1001/jamaoncol.2018.5441.

143. Sharma P, Callahan MK, Bono P, Kim J, Spiliopoulou P, Calvo E et al. Nivolumab monotherapy in recurrent metastatic urothelial carcinoma (CheckMate 032): a multicentre, open-label, two-stage,multi-arm, phase 1/2 trial. Lancet Oncol. 2016 Nov; 17 (11): 1590-1598.

144. Sharma P, Retz M, Siefker-Radtke A, Baron A, Necchi A, Bedke J et al. Nivolumab in metastatic urotelial carcinoma after platinum therapy (CheckMate 275): a multicentre, single-arm, phase 2 trial. Lancet Oncol. 2017 Mar; 18 (3): 312-322.

145. Shen J, Chang J, Mendenhall M, Cherry G, Goldman JW, Kulkarni RP. Diverse cutaneous adverse eruptions caused by anti-programmed cell death-1 (PD-1) and anti-programmed cell death ligand-1 (PD-L1) immunotherapies: clinical features and management. Ther Adv Med Oncol. 2018 Jan 22; 10: 1758834017751634.

146. Shi VJ, Rodic N, Gettinger S, Leventhal JS, Neckman JP, Girardi M et al. Clinical and Histologic Features of Lichenoid Mucocutaneous Eruptions Due to Anti-Programmed Cell Death 1 and Anti-Programmed Cell Death Ligand 1 Immunotherapy. JAMA Dermatol. 2016 Oct 1; 152 (10): 1128-1136.

147. Shimizu T, Seto T, Hirai F, Takenoyama M, Nosaki K, Tsurutani J et al. Phase 1 study of pembrolizumab (MK-3475; anti-PD-1 monoclonal antibody) in Japanese patients with advanced solid tumors. Invest New Drugs. 2016 Jun; 34 (3): 347-54.

148. Siegel J, Totonchy M, Damsky W, Berk-Krauss J, Castiglione F Jr, Sznol M et al. Bullous disorders associated with anti-PD-1 and anti-PD-L1 therapy: A retrospective analysis evaluating the clinical and histopathologic features, frequency, and impact on cancer therapy. J Am Acad Dermatol. 2018 Dec; 79 (6): 1081-1088.

149. Siu LL, Even C, Mesía R, Remenar E, Daste A, Delord JP et al. Safety and Efficacy of Durvalumab With or Without Tremelimumab in Patients With PD-L1-Low/Negative Recurrent or Metastatic HNSCC: The Phase 2 CONDOR Randomized Clinical Trial. JAMA Oncol. 2018 Nov 1. doi: 10.1001/jamaoncol.2018.4628.

150. Slovin SF, Higano CS, Hamid O, Tejwani S, Harzstark A, Alumkal JJ et al. Ipilimumab alone or in combination with radiotherapy in metastatic castration-resistant prostate cancer: results from an open-label, multicenter phase I/II study. Ann Oncol. 2013 Jul; 24 (7): 1813-21.

151. Small EJ, Tchekmedyian NS, Rini BI, Fong L, Lowy I, Allison JP. A pilot trial of CTLA-4 blockade with human anti-CTLA-4 in patients with hormone-refractory prostate cancer. Clin Cancer Res. 2007 Mar 15; 13 (6): 1810-5.

152. Strauss J, Heery CR, Schlom J, Madan RA, Cao L, Kang Z et al. Phase I Trial of M7824 (MSB0011359C), a Bifunctional Fusion Protein Targeting PD-L1 and TGFβ, in Advanced Solid Tumors. Clin Cancer Res. 2018 Mar 15; 24 (6): 1287-1295.

153. Sun D, Ma J, Wang J, Zhang F, Wang L, Zhang S et al. Clinical observation of immune checkpoint inhibitors in the treatment of advanced pancreatic cancer: a real-world study in Chinese cohort. Ther Clin Risk Manag. 2018 Sep 11; 14: 1691-1700.

154. Tahara M, Muro K, Hasegawa Y, Chung HC, Lin CC, Keam B et al. Pembrolizumab in Asia-Pacific patients with advanced head and neck squamous cell carcinoma: Analyses from KEYNOTE-012. Cancer Sci. 2018 Mar; 109 (3): 771-776.

155. Tarhini AA, Cherian J, Moschos SJ, Tawbi HA, Shuai Y, Gooding WE et al. Safety and efficacy of combination immunotherapy with interferon alfa-2b and tremelimumab in patients with stage IV melanoma. J Clin Oncol. 2012 Jan 20; 30 (3): 322-8.

156. Tarhini AA, Edington H, Butterfield LH, Lin Y, Shuai Y, Tawbi H et al. Immune monitoring of the circulation and the tumor microenvironment in patients with regionally advanced melanoma receiving neoadjuvant ipilimumab. PLoS One. 2014 Feb 3; 9 (2): e87705.

157. Taylor M, Antonia S, Bendell J, Calvo E, Jäger D, Braud F et al. Phase I/II study of nivolumab with or without ipilimumab for treatment of recurrent small cell lung cancer (SCLC): CA209-032. Journal for ImmunoTherapy of Cancer. 2015; **3 (Suppl 2):** P376.

158. Teraoka S, Fujimoto D, Morimoto T, Kawachi H, Ito M, Sato Y et al. Early Immune-Related Adverse Events and Association with Outcome in Advanced Non-Small Cell Lung Cancer Patients Treated with Nivolumab: A Prospective Cohort Study. J Thorac Oncol. 2017 Dec; 12 (12): 1798-1805.

159. Theurich S, Rothschild SI, Hoffmann M, Fabri M, Sommer A, Garcia-Marquez M et al. Local Tumor Treatment in Combination with Systemic Ipilimumab Immunotherapy Prolongs Overall Survival in Patients with Advanced Malignant Melanoma. Cancer Immunol Res. 2016 Sep 2; 4 (9): 744-54.

160. Thronicke A, Steele ML, Grah C, Matthes B, Schad F. Clinical safety of combined therapy of immune checkpoint inhibitors and Viscum album L. therapy in patients with advanced or metastatic cancer. BMC Complement Altern Med. 2017 Dec 13; 17 (1): 534.

161. Toi Y, Sugawara S, Kawashima Y, Aiba T, Kawana S, Saito R et al. Association of Immune-Related Adverse Events with Clinical Benefit in Patients with Advanced Non-Small-Cell Lung Cancer Treated with Nivolumab. Oncologist. 2018 Nov; 23 (11): 1358-1365.

162. Tomita Y, Fukasawa S, Shinohara N, Kitamura H, Oya M, Eto M et al. Nivolumab versus everolimus in advanced renal cell carcinoma: Japanese subgroup analysis from the CheckMate 025 study. Jpn J Clin Oncol. 2017 Jul 1; 47 (7): 639-646.

163. Toon CW, Hasovits C, Paik J, Field M, Chou A, Hugh TJ et al. Skin rash, a kidney mass and a family mystery dating back to World War II. Med J Aust. 2014 Jul 7; 201 (1): 58-60.

164. Topalian SL, Hodi FS, Brahmer JR, Gettinger SN, Smith DC, McDermott DF et al. Safety, activity, and immune correlates of anti-PD-1 antibody in cancer. N Engl J Med. 2012 Jun 28; 366 (26): 2443-54.

165. Tournoy KG, Thomeer M, Germonpré P, Derijcke S, De Pauw R, Galdermans D et al. Does nivolumab for progressed metastatic lung cancer fulfill its promises? An efficacy and safety analysis in 20 general hospitals. Lung Cancer. 2018 Jan; 115: 49-55.

166. Varga A, Piha-Paul S, Ott PA, Mehnert JM, Berton-Rigaud D, Morosky A et al. Pembrolizumab in patients with programmed death ligand 1-positive advanced ovarian cancer: Analysis of KEYNOTE-028. Gynecol Oncol. 2019 Feb; 152 (2) :243-250.

167. Vokes EE, Ready N, Felip E, Horn L, Burgio MA, Antonia SJ et al. Nivolumab versus docetaxel in previously treated advanced non-small-cell lung cancer (CheckMate 017 and CheckMate 057): 3-year update and outcomes in patients with liver metastases. Ann Oncol. 2018 Apr 1; 29 (4): 959-965.

168. Von Reibnitz D, Chaft JE, Wu AJ, Samstein R, Hellmann MD, Plodkowski AJ et al. Safety of combining thoracic radiation therapy with concurrent versus sequential immune checkpoint inhibition. Adv Radiat Oncol. 2018 May 9; 3 (3): 391-398.

169. Vonderheide RH, LoRusso PM, Khalil M, Gartner EM, Khaira D, Soulieres D et al. Tremelimumab in combination with exemestane in patients with advanced breast cancer and treatment-associated modulation of inducible costimulator expression on patient T cells. Clin Cancer Res. 2010 Jul 1; 16 (13): 3485-94.

170. Wang LL, Patel G, Chiesa-Fuxench ZC, McGettigan S, Schuchter L, Mitchell TC et al. Timing of Onset of Adverse Cutaneous Reactions Associated with Programmed Cell Death Protein 1 Inhibitor Therapy. JAMA Dermatol. 2018 Sep 1; 154 (9): 1057-1061.

171. Weber JS, Hodi FS, Wolchok JD, Topalian SL, Schadendorf D, Larkin J et al. Safety Profile of Nivolumab Monotherapy: A Pooled Analysis of Patients With Advanced Melanoma. J Clin Oncol. 2017 Mar; 35 (7): 785-792.

172. Weiss GJ, Waypa J, Blaydorn L, Coats J, McGahey K, Sangal A et al. A phase Ib study of pembrolizumab plus chemotherapy in patients with advanced cancer (PembroPlus). Br J Cancer. 2017 Jun 27; 117 (1): 33-40.

173. Welborn M, Kubicki SL, Garg N, Patel AB. Retrospective Chart Review of Cutaneous Adverse Events Associated with Tremelimumab in 17 Patients. Am J Clin Dermatol. 2018 Dec; 19 (6): 899-905.

174. Wilgenhof S, Corthals J, Heirman C, van Baren N, Lucas S, Kvistborg et al. Phase II Study of Autologous Monocyte-Derived mRNA Electroporated Dendritic Cells (TriMixDC-MEL) Plus Ipilimumab in Patients With Pretreated Advanced Melanoma. J Clin Oncol. 2016 Apr 20; 34 (12): 1330-8.

175. Wolchok JD, Chiarion-Sileni V, Gonzalez R, Rutkowski P, Grob JJ, Cowey CL et al. Overall Survival with Combined Nivolumab and Ipilimumab in Advanced Melanoma. N Engl J Med. 2017 Oct 5; 377 (14): 1345-1356.

176. Wolchok, JD, Thomas L, Bondarenko IN, O´Day S, Weber JS, Garbe C. Phase 3 randomized study of ipilimumab (IPI) plus dacarbazine (DTIC) vs DTIC alone as first line treatment in patients with unresectable stage III or IV melanoma. In 47th Annual Meeting of the American Society of Clinical Oncology. 2011; Proc ASCO: Abstract LBA5.

177. Yamamoto N, Nokihara H, Yamada Y, Shibata T, Tamura Y, Seki Y et al. Phase I study of Nivolumab, an anti-PD-1 antibody, in patients with malignant solid tumors. Invest New Drugs. 2017 Apr; 35 (2): 207-216.

178. Yamazaki N, Uhara H, Fukushima S, Uchi H, Shibagaki N, Kiyohara Y S et al. Phase II study of the immune-checkpoint inhibitor ipilimumab plus dacarbazine in Japanese patients with previously untreated, unresectable or metastatic melanoma. Cancer Chemother Pharmacol. 2015 Nov; 76 (5): 969-75.

179. Yang JC, Gadgeel SM, Sequist LV, Wu CL, Papadimitrakopoulou VA, Su WC et al. Pembrolizumab in Combination With Erlotinib or Gefitinib as First-Line Therapy for Advanced NSCLC With Sensitizing EGFR Mutation. J Thorac Oncol. 2019 Mar; 14 (3): 553-559.

180. Younes A, Brody J, Carpio C, Lopez-Guillermo A, Ben-Yehuda D, Ferhanoglu B et al. Safety and activity of ibrutinib in combination with nivolumab in patients with relapsed non-Hodgkin lymphoma or chronic lymphocytic leukaemia: a phase 1/2a study. Lancet Haematol. 2019 Feb; 6 (2): e67-e78.

181. Younes A, Santoro A, Shipp M, Zinzani PL, Timmerman JM, Ansell S et al. Nivolumab for classical Hodgkin's lymphoma after failure of both autologous stem-cell transplantation and brentuximab vedotin: a multicentre, multicohort,single-arm phase 2 trial. Lancet Oncol. 2016 Sep; 17 (9): 1283-94.

182. Zandberg DP, Algazi AP, Jimeno A, Good JS, Fayette J, Bouganim N et al. Durvalumab for recurrent or metastatic head and neck squamous cell carcinoma: Results from a single-arm, phase II study in patients with ≥25% tumour cell PD-L1 expression who have progressed on platinum-based chemotherapy. Eur J Cancer. 2019 Jan; 107: 142-152.

183. Zhang S, Huang X, Zhou L, Lin S. Efficacy of concurrent chemoradiotherapy combined with nimotuzumab for low-risk T4 stage nasopharyngeal carcinoma: A pilot study. Medicine (Baltimore). 2018 Sep; 97 (38): e12503.

184. Zhu AX, Finn RS, Edeline J, Cattan S, Ogasawara S, Palmer D et al. Pembrolizumab in patients with advanced hepatocellular carcinoma previously treated with sorafenib (KEYNOTE-224): a non-randomised, open-label phase 2 trial. Lancet Oncol. 2018 Jul; 19 (7): 940-952.
