# Supplementary material for: Prevalence of dermatological toxicities in patients with melanoma undergoing immunotherapy: Systematic review and meta-analysis
Source: PLoS One. 2021 Aug 6;16(8):e0255716. doi: 10.1371/journal.pone.0255716 (PMC8345892; doi:10.1371/journal.pone.0255716)
Supplement: S3 File — (DOCX) [file pone.0255716.s006.docx]

S3 File- Risk of bias in the included studies (n=39).

| **Author, Year** | **Q1** | **Q2** | **Q3** | **Q4** | **Q5** | **Q6** | **Q7** | **Q8** | **Q9** | **Total** | **Risk of bias** |
| --- | --- | --- | --- | --- | --- | --- | --- | --- | --- | --- | --- |
| ALTOMONTE et al, 2013 [30] | Y | U | U | Y | Y | Y | Y | Y | U | 66,7% | MODERATE |
| ASCIERTO et al, 2017 [49] | Y | Y | Y | Y | Y | Y | Y | Y | Y | 100% | LOW |
| CAMACHO et al, 2009 [26] | U | Y | U | Y | Y | Y | Y | Y | U | 66,7% | MODERATE |
| DIKA et al, 2017 [50] | U | U | U | U | U | Y | Y | U | U | 22,2% | HIGH |
| EGGERMONT et al, 2016 [45] | Y | Y | U | Y | Y | Y | Y | Y | U | 77,7% | LOW |
| EGGERMONT et al, 2018 [59] | Y | Y | Y | Y | Y | Y | Y | Y | Y | 100% | LOW |
| HAMID et al, 2013 [31] | U | Y | U | Y | Y | Y | Y | Y | U | 66,7% | MODERATE |
| HODI et al, 2016 [46] | Y | Y | Y | Y | Y | Y | Y | Y | Y | 100% | LOW |
| HUA et al, 2016 [47] | U | N | U | N | U | Y | Y | Y | U | 33,3% | HIGH |
| JUNG et al, 2017 [51] | U | U | U | Y | U | Y | Y | Y | Y | 55,6% | MODERATE |
| KU et al, 2010 [27] | U | Y | U | Y | U | Y | Y | Y | U | 55,5% | MODERATE |
| LARKIN et al, 2018 [60] | Y | Y | Y | Y | Y | Y | Y | Y | Y | 100% | LOW |
| LONG^a^ et al, 2017 [52] | Y | Y | Y | Y | Y | Y | Y | Y | U | 88,9% | HIGH |
| LONG^b^ et al, 2017 [53] | Y | Y | Y | Y | Y | Y | Y | Y | Y | 100% | LOW |
| MARGOLIN et al, 2012 [29] | N | Y | N | Y | Y | Y | Y | Y | Y | 77,8% | LOW |
| RUIZ-MORALES et al, 2014 [37] | N | N | N | Y | Y | N | Y | U | U | 33,3% | HIGH |
| NAKAMURA et al, 2016 [48] | U | U | U | U | U | Y | Y | Y | U | 33,3% | HIGH |
| NAMIKAWA et al, 2018 [61] | N | N | N | Y | Y | Y | Y | Y | Y | 66,7% | MODERATE |
| POSTOW et al, 2013 [32] | U | U | U | U | U | U | U | Y | U | 11,1% | HIGH |
| POSTOW et al, 2015 [39] | Y | Y | Y | Y | Y | Y | U | U | U | 66,7% | MODERATE |
| RIBAS et al, 2013 [33] | Y | Y | Y | Y | Y | Y | Y | Y | Y | 100% | LOW |
| RIBAS et al, 2015 [40] | Y | Y | Y | Y | Y | Y | Y | Y | Y | 100% | LOW |
| ROBERT et al, 2011 [28] | Y | Y | Y | Y | Y | Y | Y | Y | U | 88,9% | LOW |
| ROBERT et al, 2014 [38] | Y | Y | Y | Y | Y | Y | Y | Y | Y | 100% | LOW |
| ROBERT et al, 2015 [41] | Y | Y | Y | Y | Y | Y | Y | Y | Y | 100% | LOW |
| SHOUSHTARI et al, 2018 [62] | Y | Y | Y | Y | Y | Y | Y | Y | Y | 100% | LOW |
| SOLDATOS et al, 2018 [63] | U | U | U | Y | U | N | N | U | U | 11,1% | HIGH |
| VOSKENS et al, 2013 [34] | Y | U | Y | U | U | U | Y | U | U | 33,3% | HIGH |
| WEBER et al, 2008 [25] | Y | Y | Y | Y | Y | U | Y | U | U | 66,7% | MODERATE |
| WEBER et al, 2013 [35] | Y | Y | Y | Y | Y | Y | Y | Y | I | 88,9% | LOW |
| WEBER et al, 2017 [54] | Y | Y | Y | Y | Y | Y | Y | Y | U | 88,9% | LOW |
| WEN et al, 2017 [55] | U | U | U | Y | U | Y | Y | Y | U | 44,4% | HIGH |
| WOLCHOK et al, 2013 [36] | Y | Y | Y | Y | Y | Y | Y | Y | Y | 100% | LOW |
| YAMAZAKI et al, 2015 [42] | N | Y | N | Y | U | Y | Y | Y | U | 55,5% | MODERATE |
| YAMAZAKI^a^ et al, 2017 [56] | U | Y | U | Y | U | Y | Y | Y | U | 55,5% | MODERATE |
| YAMAZAKI^b^ et al, 2017 [57] | N | N | N | Y | Y | Y | Y | Y | Y | 66,7% | MODERATE |
| YAMAZAKI^c^ et al, 2017 [58] | N | Y | N | Y | U | Y | Y | Y | U | 55,5% | MODERATE |
| ZIMMER^a^ et al, 2015 [43] | Y | Y | Y | Y | Y | Y | Y | Y | Y | 100% | LOW |
| ZIMMER^b^ et al, 2015 [44] | Y | Y | N | Y | Y | Y | Y | Y | Y | 88,9% | LOW |

Source: Elaborated by the authors

Abbreviations: Y= Yes; N= No; U= Unclear; NA= Not/Applicable; Q= Questions.

| Q1 – Was the sample frame appropriate to address the target population? | Q6- Were valid methods used for the identification of the condition? |
| --- | --- |
| Q2- Were study participants recruited in an appropriate way? | Q7- Was the condition measured in a standard, reliable way for all participants? |
| Q3- Was the sample size adequate? | Q8- Was there appropriate statistical analysis? |
| Q4- Were the study subjects and setting described in detail? | Q9- Was the response rate adequate, and if not, was the low response rate managed  appropriately? |
| Q5- Was data analysis conducted with sufficient coverage of the identified sample? |  |
