# Supplementary material for: Prevalence of dermatological toxicities in patients with melanoma undergoing immunotherapy: Systematic review and meta-analysis
Source: PLoS One. 2021 Aug 6;16(8):e0255716. doi: 10.1371/journal.pone.0255716 (PMC8345892; doi:10.1371/journal.pone.0255716)
Supplement: S1 Protocol — (PDF) [file pone.0255716.s007.pdf]

Systematic review

1. * Review title.

Give the working title of the review, for example the one used for obtaining funding. Ideally the title should state succinctly the interventions or exposures being reviewed and the associated health or social problems. Where appropriate, the title should use the PI(E)COS structure to contain information on the Participants, Intervention (or Exposure) and Comparison groups, the Outcomes to be measured and Study designs to be included.

Prevalence of dermatological toxicities in cancer patients submitted to treatment with immune checkpoint inhibitors: systematic review and meta-analysis

2. Original language title.

For reviews in languages other than English, this field should be used to enter the title in the language of the review. This will be displayed together with the English language title.

Prevalência de toxicidade dermatológica em pacientes com câncer submetidos a tratamento com inibidores imunes de checkpoint: revisão sistemática e metabolise

3. * Anticipated or actual start date.

Give the date when the systematic review commenced, or is expected to commence.

31/01/2018

4. * Anticipated completion date.

Give the date by which the review is expected to be completed.

31/08/2019

5. * Stage of review at time of this submission.

Indicate the stage of progress of the review by ticking the relevant Started and Completed boxes. Additional information may be added in the free text box provided.
Please note: Reviews that have progressed beyond the point of completing data extraction at the time of initial registration are not eligible for inclusion in PROSPERO. Should evidence of incorrect status and/or completion date being supplied at the time of submission come to light, the content of the PROSPERO record will be removed leaving only the title and named contact details and a statement that inaccuracies in the stage of the review date had been identified.
This field should be updated when any amendments are made to a published record and on completion and publication of the review. If this field was pre-populated from the initial screening questions then you are not able to edit it until the record is published.

The review has not yet started: No

Review stage	Started	Completed
Preliminary searches	Yes	Yes
Piloting of the study selection process	Yes	No
Formal screening of search results against eligibility criteria	No	No
Data extraction	No	No
Risk of bias (quality) assessment	No	No
Data analysis	No	No

Provide any other relevant information about the stage of the review here (e.g. Funded proposal, protocol not yet finalised).

6. * Named contact.

The named contact acts as the guarantor for the accuracy of the information presented in the register record.

Christiane Inocêncio Vasques

Email salutation (e.g. "Dr Smith" or "Joanne") for correspondence:

Professor Christiane Vasques

7. * Named contact email.

Give the electronic mail address of the named contact.

chvasques@unb.br

8. Named contact address

Give the full postal address for the named contact.

University of Brasilia - School of Health Science - Nursing Department

Campus Universitário Darcy Ribeiro, Asa Norte, Brasília-DF, Brazil, CEP: 70910-900

9. Named contact phone number.

Give the telephone number for the named contact, including international dialling code.

+55(61)98121-4440

10. * Organisational affiliation of the review.

Full title of the organisational affiliations for this review and website address if available. This field may be completed as 'None' if the review is not affiliated to any organisation.

University of Brasília

Organisation web address:

www.unb.br

11. * Review team members and their organisational affiliations.

Give the title, first name, last name and the organisational affiliations of each member of the review team. Affiliation refers to groups or organisations to which review team members belong.

Professor Christiane Inocêncio Vasques. University of Brasília
Professor Paula Elaine Diniz dos Reis. University of Brasilia
Professor Elaine Barros Ferreira. University of Brasilia
Dr Giovanni Damiani. Policlinic of Milan
Miss Nathali Felicia Mineiro dos Santos Garrett. University of Brasilia
Dr Ana Cristina Carvalho da Costa. Department of Health of the Federal District

12. * Funding sources/sponsors.

Give details of the individuals, organizations, groups or other legal entities who take responsibility for initiating, managing, sponsoring and/or financing the review. Include any unique identification numbers assigned to the review by the individuals or bodies listed.

None

13. * Conflicts of interest.

List any conditions that could lead to actual or perceived undue influence on judgements concerning the main topic investigated in the review.

None

None specified.

14. Collaborators.

Give the name and affiliation of any individuals or organisations who are working on the review but who are not listed as review team members.

15. * Review question.

State the question(s) to be addressed by the review, clearly and precisely. Review questions may be specific or broad. It may be appropriate to break very broad questions down into a series of related more specific questions. Questions may be framed or refined using PI(E)COS where relevant.

What is the prevalence of dermatological toxicities in cancer patients undergoing treatment with immune checkpoints inhibitors?

16. * Searches.

State the sources that will be searched. Give the search dates, and any restrictions (e.g. language or publication period). Do NOT enter the full search strategy (it may be provided as a link or attachment.)

We will search the following electronic bibliographic databases: MEDLINE, LILACS (Literature of Latin America and Caribe in Health Science), LIVIVO, The Cochrane Library, Web of Science (Science and Social Science Citation Index), Scopus and CINAHL (Cumulative Index to Nursing and Allied Health).

We will include observational and clinical trials studies which the population of interest are cancer patients submitted to treatment with targeted therapies and/or immune checkpoint inhibitors.

The studies must include reference to the prevalence of dermatological toxicity, without time or language restrictions.

17. URL to search strategy.

Give a link to a published pdf/word document detailing either the search strategy or an example of a search strategy for a specific database if available (including the keywords that will be used in the search strategies), or upload your search strategy.Do NOT provide links to your search results.

Alternatively, upload your search strategy to CRD in pdf format. Please note that by doing so you are consenting to the file being made publicly accessible.

Do not make this file publicly available until the review is complete

18. * Condition or domain being studied.

Give a short description of the disease, condition or healthcare domain being studied. This could include health and wellbeing outcomes.

Dermatological toxicity is the first to occur in patients undergoing treatment with immune checkpoint inhibitors. In many cases, due to the severity of the skin reactions and the impact on patient's quality of life, it may require dose reduction or discontinuation of treatment. In this review, it will be considered as dermatological toxicity: skin rashes, lichenoid reactions, psoriasis, Grover's disease, pruritus, vitiligo, autoimmune skin disorders, bullous pemphigoide, acneiform rash and papulopustular rosacea, papulopustular eruption, xerosis and related disorders, cutaneous adnexal reactions such as paronychia, trichomegaly, splinter hemorrhages, telogen effluvium, alopecia, acneiform reactions, multikinase induced maculopapular rash, cutaneous vasculitis, hand-foot reaction, skin pigmentation alterations such as yellowish discoloration, hypopigmentation, perifollicular pigmentation.

19. * Participants/population.

Give summary criteria for the participants or populations being studied by the review. The preferred format includes details of both inclusion and exclusion criteria.

Adult cancer patients submitted to treatment with immune checkpoints inhibitors, exclusive or associated with chemotherapy and/or radiotherapy. It will be included studies that present the prevalence of dermatological toxicity.

20. * Intervention(s), exposure(s).

Give full and clear descriptions or definitions of the nature of the interventions or the exposures to be reviewed.

Dermatological toxicity related to immune checkpoints inhibitors.

21. * Comparator(s)/control.

Where relevant, give details of the alternatives against which the main subject/topic of the review will be compared (e.g. another intervention or a non-exposed control group). The preferred format includes details of both inclusion and exclusion criteria.

None.

22. * Types of study to be included.

Give details of the types of study (study designs) eligible for inclusion in the review. If there are no

restrictions on the types of study design eligible for inclusion, or certain study types are excluded, this should be stated. The preferred format includes details of both inclusion and exclusion criteria.

Clinical trials, Observational studies.

23. Context.

Give summary details of the setting and other relevant characteristics which help define the inclusion or exclusion criteria.

24. * Main outcome(s).

Give the pre-specified main (most important) outcomes of the review, including details of how the outcome is defined and measured and when these measurement are made, if these are part of the review inclusion criteria.

Prevalence of dermatological toxicity

Timing and effect measures

CTCAE

25. * Additional outcome(s).

List the pre-specified additional outcomes of the review, with a similar level of detail to that required for main outcomes. Where there are no additional outcomes please state 'None' or 'Not applicable' as appropriate to the review

Prevalence of dermatological toxicities caused by concomitant use of chemotherapy and immune checkpoint inhibitors;

Prevalence of dermatological toxicities caused by the concomitant use of radiotherapy and immune checkpoint inhibitors;

Prevalence of dermatological toxicities caused by concomitant use of two or more immune checkpoint inhibitors.

Timing and effect measures

None

26. * Data extraction (selection and coding).

Describe how studies will be selected for inclusion. State what data will be extracted or obtained. State how this will be done and recorded.

The selection will be completed in two phases. In phase 1, two reviewers independently will review the titles and abstracts of all identified electronic database citations. A third author will be involved when required to make a final decision. Any studies that appeared not to fulfill the inclusion criteria will be discarded. In phase 2, the same selection criteria will be applied to the full articles to confirm their eligibility. The same two reviewers independently will participate in phase 2. The references list of all included articles will be reviewed by one examiner. The articles selected will be read by both examiners. Any disagreement in either phase will be resolved by discussion and mutual agreement between the three reviewers. Final selection will be always based on the full-text of the publication.

27. * Risk of bias (quality) assessment.

Describe the method of assessing risk of bias or quality assessment. State which characteristics of the studies will be assessed and any formal risk of bias tools that will be used.

JBI Critical Appraisal Checklist for Studies Reporting Prevalence Data will be used to asses the quality of the included studies.

28. * Strategy for data synthesis.

Provide details of the planned synthesis including a rationale for the methods selected. This **must not be generic text** but should be **specific to your review** and describe how the proposed analysis will be applied to your data.

We will provide a narrative synthesis of the findings from the included studies, structured around the type of treatment, the target population characteristics, and the type and prevalence of hematological toxicity.

We will also provide a quantitative synthesis if the included studies are sufficiently homogenous.

29. * Analysis of subgroups or subsets.

State any planned investigation of 'subgroups'. Be clear and specific about which type of study or participant will be included in each group or covariate investigated. State the planned analytic approach.

This is a qualitative synthesis and while subgroup analyses may be undertaken it is not possible to specify the groups in advance.

30. * Type and method of review.

Select the type of review and the review method from the lists below. Select the health area(s) of interest for your review.

Type of review

Cost effectiveness
No

Diagnostic
No

Epidemiologic
Yes

Individual patient data (IPD) meta-analysis
No

Intervention
No

Meta-analysis
No

Methodology
No

Narrative synthesis
No

Network meta-analysis
No

Pre-clinical
No

Prevention
No

Prognostic

No

Prospective meta-analysis (PMA)
No

Review of reviews
No

Service delivery
No

Synthesis of qualitative studies
No

Systematic review
Yes

Other
No

Health area of the review

Alcohol/substance misuse/abuse
No

Blood and immune system
No

Cancer
Yes

Cardiovascular
No

Care of the elderly
No

Child health
No

Complementary therapies
No

Crime and justice
No

Dental
No

Digestive system
No

Ear, nose and throat
No

Education
No

Endocrine and metabolic disorders
No

Eye disorders
No

General interest
No

Genetics
No

Health inequalities/health equity
No

Infections and infestations

No

International development
No

Mental health and behavioural conditions
No

Musculoskeletal
No

Neurological
No

Nursing
No

Obstetrics and gynaecology
No

Oral health
No

Palliative care
No

Perioperative care
No

Physiotherapy
No

Pregnancy and childbirth
No

Public health (including social determinants of health)
No

Rehabilitation
No

Respiratory disorders
No

Service delivery
No

Skin disorders
Yes

Social care
No

Surgery
No

Tropical Medicine
No

Urological
No

Wounds, injuries and accidents
No

Violence and abuse
No

31. Language.

Select each language individually to add it to the list below, use the bin icon to remove any added in error.
English

There is not an English language summary

32. Country.

Select the country in which the review is being carried out from the drop down list. For multi-national collaborations select all the countries involved.

Brazil

33. Other registration details.

Give the name of any organisation where the systematic review title or protocol is registered (such as with The Campbell Collaboration, or The Joanna Briggs Institute) together with any unique identification number assigned. (N.B. Registration details for Cochrane protocols will be automatically entered). If extracted data will be stored and made available through a repository such as the Systematic Review Data Repository (SRDR), details and a link should be included here. If none, leave blank.

34. Reference and/or URL for published protocol.

Give the citation and link for the published protocol, if there is one

Give the link to the published protocol.

Alternatively, upload your published protocol to CRD in pdf format. Please note that by doing so you are consenting to the file being made publicly accessible.

No I do not make this file publicly available until the review is complete

Please note that the information required in the PROSPERO registration form must be completed in full even if access to a protocol is given.

35. Dissemination plans.

Give brief details of plans for communicating essential messages from the review to the appropriate audiences.

Do you intend to publish the review on completion?

Yes

36. Keywords.

Give words or phrases that best describe the review. Separate keywords with a semicolon or new line. Keywords will help users find the review in the Register (the words do not appear in the public record but are included in searches). Be as specific and precise as possible. Avoid acronyms and abbreviations unless these are in wide use.

Dermatological toxicity; Skin toxicity; Immune checkpoint inhibitors

37. Details of any existing review of the same topic by the same authors.

Give details of earlier versions of the systematic review if an update of an existing review is being registered, including full bibliographic reference if possible.

38. * Current review status.

Review status should be updated when the review is completed and when it is published. For newregistrations the review must be Ongoing.
Please provide anticipated publication date

Review_Ongoing

39. Any additional information.

Provide any other information the review team feel is relevant to the registration of the review.

40. Details of final report/publication(s).

This field should be left empty until details of the completed review are available.

Give the link to the published review.
