# Supplementary material for: Prevalence of dermatological toxicities in patients with melanoma undergoing immunotherapy: Systematic review and meta-analysis
Source: PLoS One. 2021 Aug 6;16(8):e0255716. doi: 10.1371/journal.pone.0255716 (PMC8345892; doi:10.1371/journal.pone.0255716)
Supplement: Attachment — Submitted filename: Plos one.docx [file pone.0255716.s008.docx]

Title

1. Make clear in the Title that the population was not “cancer” patients but “melanoma” patients.

**Abstract**:

1. The authors mention “To identify the prevalence of cutaneous toxicity in patients with melanoma on treatment with immune isolated checkpoint inhibitors, combined or associated with chemotherapy and/or radiotherapy.”

Did you consider checkpoint inhibitors as monotherapy AND combined with chemo and radiotherapy, or did you consider only the combinations? Please clarify.

**Introduction**

1. S64 : has -> have
2. S72: differente -> different

**Material methods**

1. PRISMA: OK
2. PROSPERO: OK
3. Inclusion και exclusion criteria: clear and analytic
4. Search strategy – study selection – data extraction: OK
5. S145: omit parenthesis
6. Risk of bias: ΟΚ
7. Summary and synthesis of the results: the authors mention the primary outcome and define it (159: «the frequence … ICI”), but in synthesis they do not mention how did they estimate the prevalence (is it pooled prevalence?). Furthermore they do not mention if they estimated confidence intervals, prevalence and CI, especially, in the cases of very low incidence (logit or double arcsine transformation method is preferable, Barendregt J et al. Meta-analysis of prevalence). In my view, even if it was not performed, it should be mentioned in the statistical analysis section.
8. **Two major issues**:

- Initially, the authors mention they estimated heterogeneity (I2) and based on this the used the corresponding models (random ή fixed), but they do not analyze the reasons of heterogeneity, not even in the section they present their results (s302 – 308). In addition they do not mention if there were subgroup analyses for the investigation of heterogeneity, especially when found very high (Ι2 = 88%. Ι2= 84%). Even if not performed (or when performed did not change the result?), it would be interesting to know which was the conclusion.
- There are no comments or test of publication bias (no funnel plot). This must be included.

**Results**

1. Flow chart – study characteristics: OK
2. S224-5: reason for not analyzed the antiPDL1. It is fine that the authors mention that they did not study this class of drugs, but I would like them to give reasons for this decision.
3. Graphic s4. In the text the authors mention that pruritus 3-4 is 1% and eruption 3-4 is 1% with reference to s4. However, in s4 the authors have included only pruritus.
4. Graphic s4. Which was the methodology the authors used for the investigation of heterogeneity among the diverse studies for pruritus and the eruption? Was it random or fixed? Please clarify. If they did so, exclusively for pruritus, they must change s315.
5. S394 differente -> different

**General comments**

1. Page 13,14 (s 230 – 263) the authors give the rates of AE only as a range. The latter was not pre-defined in the statistical analysis section. I presume these are the CIs, but I consider they should be defined for clarification reasons.
2. In addition, they give just the range, without a certain rate in conjunction with the range.
3. In the end of each paragraph (e.g. 234-235 or 246-247 or 257-259, there are no rates and CIs, but only their conclusions. I recommend to include them, too.
