# Supplementary material for: Prevalence of dermatological toxicities in patients with melanoma undergoing immunotherapy: Systematic review and meta-analysis
Source: PLoS One. 2021 Aug 6;16(8):e0255716. doi: 10.1371/journal.pone.0255716 (PMC8345892; doi:10.1371/journal.pone.0255716)
Supplement: Attachment — Submitted filename: Response to reviewers PONE-D-19-20870.docx [file pone.0255716.s009.docx]

October 14^th^, 2020

Gayle E. Woloschak, PhD
Academic Editor
PLOS ONE

Dear Dr. Woloschak,

Thank you for the opportunity to revise and improve the manuscript PONE-D-19-20870 entitled " Prevalence of dermatological toxicities in cancer patients undergoing immunotherapy: systematic review and meta-analysis".

We would like to thank the reviewers for their valuable comments and suggestions. We have addressed all points raised by the reviewers and made revisions accordingly. Below, we have listed the reviewer’s comments and our responses. The changes have been highlighted in the revised version and marked in red font.

Responses to the Reviewers

Title

1. Make clear in the Title that the population was not “cancer” patients but “melanoma” patients.

**Response:** We replaced the word “cancer” for “melanoma”. The modified title is *“Prevalence of dermatological toxicities in patients with melanoma undergoing immunotherapy: systematic review and meta-analysis”*

**Abstract**:

1. The authors mention “To identify the prevalence of cutaneous toxicity in patients with melanoma on treatment with immune isolated checkpoint inhibitors, combined or associated with chemotherapy and/or radiotherapy.”

Did you consider checkpoint inhibitors as monotherapy AND combined with chemo and radiotherapy, or did you consider only the combinations? Please clarify.

**Response:** It was clarified in the abstract and at the end of introduction.

*"To identify the prevalence of cutaneous toxicity in patients with melanoma on treatment with immune checkpoint inhibitors as monotherapy and/or, combined with chemotherapy and/or radiotherapy" (page 2, line 38; page 4, line 82).*

**Introduction**

1. S64 : has -> have
2. S72: differente -> different

**Response:** Thank you. We replaced the words.

**Material methods**

1. S145: omit parenthesis

**Response:** Done.

2. Summary and synthesis of the results: the authors mention the primary outcome and define it (159: «the frequence … ICI”), but in synthesis they do not mention how did they estimate the prevalence (is it pooled prevalence?). Furthermore, they do not mention if they estimated confidence intervals, prevalence and CI, especially, in the cases of very low incidence (logit or double arcsine transformation method is preferable, Barendregt J et al. Meta-analysis of prevalence). In my view, even if it was not performed, it should be mentioned in the statistical analysis section.

**Response:** Thank you for this appointment. The prevalence was estimated by the number of events out of the total of the sample. We add this information in the statistical analysis section (page 7, line 164).

3. **Two major issues**:

- Initially, the authors mention they estimated heterogeneity (I2) and based on this the used the corresponding models (random ή fixed), but they do not analyze the reasons of heterogeneity, not even in the section they present their results (s302 – 308). In addition, they do not mention if there were subgroup analyses for the investigation of heterogeneity, especially when found very high (Ι2 = 88%. Ι2= 84%). Even if not performed (or when performed did not change the result?), it would be interesting to know which was the conclusion.

**Response:** We added more information to the Method Section (page 7, lines 170-172). In the synthesis of the results, we add more details to the outcomes, such as the p value and CI for each subgroup meta-analysis (page 31, lines 317-323).

- There are no comments or test of publication bias (no funnel plot). This must be included.

**Response:** We performed the funnel plot and doi plot for each outcome. However, Furuya-kanamori et al (2018) recommend that funnel plot should not be considered, since in prevalence studies the graphic results could be noninterpretability. Based on this paper, we did not presented the funnel plot and doi plot. We added this information in the method section (page 7, lines 170 -172).

**Results**

1. S224-5: reason for not analyzed the antiPDL1. It is fine that the authors mention that they did not study this class of drugs, but I would like them to give reasons for this decision.

**Response:** Some studies which evaluated anti-PDL1 drugs were identified in the database search. However, they were excluded on phase 2 for the following reasons (as mentioned in the Appendix 2 - S2 file): absence of dermatological toxicity, complementary studies with duplicated data, data extraction not possible and, other types of cancer.

2. Graphic s4. In the text the authors mention that pruritus 3-4 is 1% and eruption 3-4 is 1% with reference to s4. However, in s4 the authors have included only pruritus.

**Response:** We replaced the S4 figure which now presents the results for the rash grade 3-4.

3. Graphic s4. Which was the methodology the authors used for the investigation of heterogeneity among the diverse studies for pruritus and the eruption? Was it random or fixed? Please clarify. If they did so, exclusively for pruritus, they must change s315.

**Response:** Thank you for this important comment about the meta-analysis effect. We used only random effect and we changed the figure.

4. S394 differente -> different

**Response:** Done.

**General comments**

1. Page 13,14 (s 230 – 263) the authors give the rates of AE only as a range. The latter was not pre-defined in the statistical analysis section. I presume these are the CIs, but I consider they should be defined for clarification reasons.

**Response:** The range are not based on the CIs, it is the frequency of the event, i.e. the percentage of the events out of the total n.

2. In addition, they give just the range, without a certain rate in conjunction with the range.

**Response:** As those symptoms were secondary outcomes in most individual studies, it was only possible to extract the percentage of the number of events of the AE.

3. In the end of each paragraph (e.g. 234-235 or 246-247 or 257-259, there are no rates and CIs, but only their conclusions. I recommend to include them, too.

**Response:** We included all the rates in the conclusion sentence made in each paragraph (page 13, lines 226-228; page 14, lines 245-248; page 14, lines 259-271; page 15, lines 276-277).
