# Supplementary material for: Prevalence of dermatological toxicities in patients with melanoma undergoing immunotherapy: Systematic review and meta-analysis
Source: PLoS One. 2021 Aug 6;16(8):e0255716. doi: 10.1371/journal.pone.0255716 (PMC8345892; doi:10.1371/journal.pone.0255716)
Supplement: Attachment — Submitted filename: 16 03 2020 Response to reviewers PONE-D-19-20870.docx [file pone.0255716.s010.docx]

March 16^th^, 2021

Gayle E. Woloschak, PhD
Academic Editor
PLOS ONE

Dear Dr. Woloschak,

Thank you for the opportunity to revise and improve the manuscript PONE-D-19-20870R1 entitled " Prevalence of dermatological toxicities in cancer patients undergoing immunotherapy: systematic review and meta-analysis".

We would like to thank the reviewers for their valuable comments and suggestions. We have addressed all points raised by the reviewers and made revisions accordingly. Below, we have listed the reviewer’s comments and our responses. The changes have been highlighted in the revised version and marked in red font.

Responses to the Reviewers

**Reviewer #3:**

Overview: Important meta-analysis on a very common toxicity seen with ICIs. However I have some concerns about the way the information is presented. It reads a bit confusing/complicated. Another general important concept to address is WHY cutaneous toxicity in melanoma patients getting ICI is of specific interest. Notably vitiligo. Arguably the rash and vitiligo are a type of on target tox since melanoma starts as cancer of skin and attach of melanocytes suggests on target mechanism. Further vitiligo has correlated with response/survival in melanoma. In addition, similar to endocrinopathies, cutaneous tox is one of the irAEs that you can often treat through and not hold treatment but just manage symptomatically - unless progresses to SJS or TEN. Though not included in the meta-analysis I would add a paragraph just listing what has been reported in the literature when it comes to types of rashes (maculo-papular, psoriatic like, lichen planus, pemphigous etc).
**Response:** As suggested by the reviewer, we added on discussion section a paragraph listing other dermatological toxicities mentioned by the studies included in the review. *“Other dermatological manifestations were identified in the studies included in this review such as maculopapular exanthema, erythema multiforme, dermatitis, acneiform rash, lichenoid exanthema, folliculitis, rosacea, eczema, leukoderma, seborrheic dermatitis, and alopecia. The majority of them were classified as grade 1 and 2. Although it is possible to manage these AEs in most cases, early identification plays a key role in the prevention of severe cases, avoiding treatment interruption.”* (page 32, lines 408-413)

**Abstract:**
Page 2 Line 45 - The first thing that jumped out to be was that the overall prevalence of pruritis (17%) and rash (12%) seem VERY low. I think there may be issue here with how this was arrived at. If you go to the the table 2, any grade or grade 1-2 are often higher than this. For example in your TABLE when listing reference 53 which is LONG et al JAMA Oncology 2017 if you go to the original text there is a "skin tox" listed as irAE which needs to be included and then your 11% would be more like 50%+. Further unsure why here just selected the non-TBP group.

**Response:** We decided not to present the overall prevalence for pruritus and rash. These signs/symptoms are adverse events that evolve gradually, for example, the patient that presents pruritus classified as grade 1 and 2 in the beginning of the treatment can evolve to grade 3 and 4 later. Thus, when calculating the overall prevalence we may be counting the same patient twice. For this reason, we decided to present only the results of the subgroup analysis grade 1 and 2 and, grade 3 and 4 for both pruritus and rash.

The modified sentence on the abstract is : ***“****The results suggest that the most prevalent side effect was grade 1 and 2 pruritus (24%), followed by grade 1 and 2 rash (21%) and grade 1 and 2 vitiligo (10%).”* (page 2, lines 44-46)

Regarding the reference n. 53 (Long et al, 2017), which now is n. 54 due to the insertion of a new reference in the introduction, we inform that we did not selected one specific group. The data from both non-TBP group and TBP group have already been described in table. As suggested by the reviewer, we included the data related to “skin tox” in table 1.

**Introduction:**
Page 3 Line 63. Would reword sentence to read "The side effects related to ICIs are labeled as immune related adverse events (irAEs) and are thought to be related to [insert mechanism hypothesis]. Then would add something like "Though one can see irAes involving all body systems, cutaneous toxicity is of particular interest".

**Response:** We inserted the reviewer’s suggestion. The modified sentence is presented above.

*“(…) The side effects related to ICIs are labeled as immune-related adverse events (irAEs) and are thought to be related to the inflammatory response caused in several organs due to the stimulation of the immune system, especially of T cells [9,10, 11]. Though one can see irAEs involving all body systems, cutaneous toxicity is of particular interest.”* (page 3, lines 63-67)

Page 3 Like 69: Would expand this to address what the cost is related to.
**Response:** We included the information as suggested.

*“The cost associated with the management of dermatological toxicity in patients with metastatic melanoma reaches US $ 21,726.00 per month, which represent the total adjudicated amount paid to all providers for inpatient and outpatient services and drugs [14].”* (page 4, lines 77-79)

**Eligibility**
Page 4 Line 93: Would remind readers that this is MELANOMA patients.

**Response:** We included the word melanoma.

*“We included clinical trials (randomized and non-randomized) and observational studies that evaluated melanoma cancer patients undergoing treatment with a single ICI, a combination of ICIs, or a combination of an ICI with chemotherapy and/or radiotherapy and that described the prevalence of dermatological toxicity.”* (page 5, lines 105)

Study Selection
Page 6 Line 125: Would replace argumentation with discussion

Response: We replaced the word argumentation for discussion.

*“(…) Any disagreements in the first or second phase were resolved by discussion until an agreement was reached between the two authors.(…)”* (page 6, line 136)

Table 1 and 2: This needs to be simplified. There is redundancy in how reported and too many words. You could consolidate to one table with columns listing Study Author and Year / Study Design [also include line of therapy here] / Drugs tested / Dermatologic Toxicities. Purpose of this paper is to highlight derm tox, other info not needed. Also I think the percentages of derm toxicities for some of the studies is not correct. See above concern about study [53].

**Response:** We consolidated the tables. Table 2 now is presented as table 1 in which we inserted the columns author/year/contry and, study design that were originally from table 1. All other data have not been changed. Regarding the study mentioned (reference n. 53, that now is 54 because of the insertion of one reference in the introduction), the percentages are correct. We inserted only the acronymun TBP (treatment beyond progression) and non-TBP to be the same as described by the author in the article, but all the data about non-TBP and TBP groups had already been described since the first version of the manuscript. (page 13)

| **STUDY CHARACTERISTICS** | | | | **SAMPLE CHARACTERISTICS** | | | | **EXPOSURE CHARACTERISTICS** | | | | | | **OUTCOME CHARACTERISTICS** | | | | |
| --- | --- | --- | --- | --- | --- | --- | --- | --- | --- | --- | --- | --- | --- | --- | --- | --- | --- | --- |
| **Author, Year**  **Country** | | **Study design** | | **Sample size (n)** | | **Age in years (mean and range)** | | **Drug (dose and schedule)** | | **Duration of treatment** | | **Follow-up** | | **Dermatological toxicities (n) %** | | | **Main Conclusions** | |
| LONG^a^ et al, 2017 [54]  Australia | Retrospective OS | | 306  Non-beyond  progression  (221)  Beyond  progression  (85) | | 62 (18-90) | | Nivolumab (3mg/kg every 2 weeks) | | Until progression or unacceptable toxic effects. | | Not described | | Non-treatment beyond  Progression (Non-TBP)  Any Grade  Skin adverse event: (58) 26%  Pruritus: (25) 11%  Rash: (23) 10%  Grade 3 or 4  Skin adverse event: (2) 1%  Pruritus: (1) <1%  Rash: 0 | | Treatment Beyond  Progression (TBP)  Any Grade  Skin adverse event: (43) 51%  Pruritus: (23) 27%  Rash: (23) 27%  Grade 3 or 4  Skin adverse event: (1) 1%  Pruritus: 0  Rash: 0 | Patients treated beyond  their first disease progression can experience a tumor response with continued nivolumab treatment, with a safety profile consistent with that observed in patients who did not receive further treatment. | |  |

Discussion
Page 32 Lines 322-329. This is contradictory. Say mechanism unknown but then list a mechanism. Would rephrase.

**Response:** We rewrote the sentence. The modified sentence is *“Cutaneous AEs are the first toxicities to occur with the use of ICIs. Despite being self-limited, these toxicities may lead to ICI dose reduction and treatment discontinuation [66]. Dermatological toxicities might be mediated by a shared antigen which is coexpressed by the tumor cells and dermoepidermal junction [10, 12].”*

Overall would reword discussion. It is currently a repeat of what is already noted. Would comment on what is mentioned above in overview section.

**Response:** We reworded many aspects of the discussion in order to address what was mentioned in the overview section. All the changes we made are highlighted in red font.
