# Supplementary material for: Prevalence of dermatological toxicities in patients with melanoma undergoing immunotherapy: Systematic review and meta-analysis
Source: PLoS One. 2021 Aug 6;16(8):e0255716. doi: 10.1371/journal.pone.0255716 (PMC8345892; doi:10.1371/journal.pone.0255716)
Supplement: Attachment — Submitted filename: Response to reviewers PONE-D-19-20870 (2021 05 28).docx [file pone.0255716.s011.docx]

May 28^th^, 2021

Gayle E. Woloschak, PhD
Academic Editor
PLOS ONE

Dear Dr. Woloschak,

Thank you for the opportunity to revise and improve the manuscript PONE-D-19-20870R1 entitled " Prevalence of dermatological toxicities in cancer patients undergoing immunotherapy: systematic review and meta-analysis".

We would like to thank the reviewers for their valuable comments and suggestions. We have addressed all points raised by the reviewers and made revisions accordingly. Below, we have listed the reviewer’s comments and our responses. The changes have been highlighted in the revised version and marked in red font.

Responses to the Reviewers

**Reviewer #3:**

I still think you miss the opportunity to make this a more complete meta-analysis of dermatologic toxicities. As it stand right now it is mostly a summary of the incidence of "rash" "pruritis" and "vitiligo". Though as you can see a number of studies are more detailed in their descriptions. You hit on this in the conclusion a bit but the majority of text just summarizes incidence of "rash" and "pruritis" which is very common and actually in clinical practice not always clinically significant. It is the more specific toxicities such as pemphigus, EN, etc that you actually hold or stop drug.

**Response:** As suggested by the reviewer, we looked again at the studies included in this review to bring out the dermatological toxicities in more detail, which can be seen in Table 1 (page 10-20), and in the results section (pages 22 -24, lines 247 - 317).

We identifiy that erythema multiforme, skin hypopigmentation, dry skin and DRESS syndrome were also reported by two or more studies. So we reported this in the result section (pages 22 -24, lines 247 - 317). For these reason, we rerun the meta-analysis for rash, pruritus and vitiligo, and we run the meta-analysis for erythema multiforme and dry skin, now on Jamovi software (page 25-26, lines 349 - 358; 376-390). It was not possible to run the meta-analysis for skin hypopigmentation because the studies did not classified this toxicity. Among the studies that reported DRESS syndrome, one reported the toxicity as grade 1and 2, and the other one as grade 3 and 4, which made impossible to perform the meta-analysis.

We also inserted a paragraph on discussion regard the inclusion of erythema multiforme and dry skin meta-analysis: *“Erythema multiforme (EM) is not a very commom AE observed in patients undergoing immunotherapy. However, there are some case reports showing the development of EM-related to the administration of nivolumab and ipilimumab [75, 76] that required immunotherapy discontinuation, and corticosteroid treatment. Nevertheless, the studies in this review reported grade 1 and 2 EM which did not lead to treatment discontinuation. Meantime, dry skin is a commom AE related to both chemotherapy and immunotherapy. It is important to educate patients on the use of moisturizers to prevent itching”* (page 28, lines 427-433).

Regarding the more specific toxicities with clinical impact, only one case of pemphigus (long et al, 2017), Steve-Johnson syndrome (Dika et al., 2017), and EN (Ascierto et al., 2017) were reported by the studies, but the authors did not make clear if these toxicities led to treatment discontinuation. However, we had already included a paragraph in the discussion section reporting the data about Steve-Johnson syndrome identified in this review, and stating the importance of monitoring signs and symptoms indicative of Steve-Johnson syndrome and toxic epidermal necrolisis. Now, we included a sentence about the data we found about pemphigoid lesion (page 29, lines 463-465):

“*High-degree AEs may be potentially fatal; thus, patients should be carefully evaluated for symptoms consistent with Stevens-Johnson syndrome, pemphigus or toxic epidermal necrolysis [81]. In our review, one study identified Stevens-Johnson syndrome [52], classified as grade 4, affecting only one patient. Pemphigoid lesion grade 4 was also reported by one study [55], affecting only one patient. Though was not possible to perceive wheter these toxicities led to treatment discontinuation. However, toxic epidermal necrolysis was not listed in any of the studies. Both events are considered dermatological emergencies with high morbidity power which require an immediate intervention, hospitalization, and treatment discontinuation [82].”*

**Reviewer #3:**

I would maybe like to see the paper organized by type of skin toxicity as opposed to results of individual studies. A number of the studies detail other more clinical significant cutaneous toxicities. The Table is still a bit long and too detailed. Would remove the column on duration of treatment as well as main conclusion (purpose of paper is not to educate on outcomes of the studies as pertains to cancer effect).

**Response:** We are now presenting the results by type of skin toxicity (pages 22 -24, lines 247-317).

As suggested, we removed the column on duration of treatment and main conclusion from table 1.
