# Supplementary material for: Prevalence of dermatological toxicities in patients with melanoma undergoing immunotherapy: Systematic review and meta-analysis
Source: PLoS One. 2021 Aug 6;16(8):e0255716. doi: 10.1371/journal.pone.0255716 (PMC8345892; doi:10.1371/journal.pone.0255716)
Supplement: Attachment — Submitted filename: Response to reviewers PONE-D-19-20870 (2021 07 12).docx [file pone.0255716.s012.docx]

July 12^th^, 2021

Gayle E. Woloschak, PhD
Academic Editor
PLOS ONE

Dear Dr. Woloschak,

Thank you for the opportunity to revise and improve the manuscript PONE-D-19-20870R1 entitled " Prevalence of dermatological toxicities in cancer patients undergoing immunotherapy: systematic review and meta-analysis".

We would like to thank the reviewers for their valuable comments and suggestions. We have addressed all points raised by the reviewers and made revisions accordingly. Below, we have listed the reviewer’s comments and our responses. The changes have been highlighted in the revised version and marked in red font.

Responses to the Reviewers

**Reviewer #3:**

1) Page 3 Line 55: Would reword - patients achieving responses vs delay of disease progression are two different outcomes. May say something like "Many patients have been living longer due to remarkable responses and delay of disease progression"

**Our response:** We reworded the sentence as suggested by the reviewer: *“Many patients have been living longer due to remarkable responses and delay of disease progression during ICI treatment [3].*”

r

**Reviewer #3:**

2) Page 3 Line 63: Would reword - "Although in many cancers one can see anti-tumor activity with ICIs and traditional chemotherapy, the types, mechanisms, and rates of side effects differ"

**Our response:** We reworded the sentence as suggested by the reviewer: *“Although in many cancers one can see anti-tumor activity with ICIs and traditional chemotherapy, the types, mechanisms, and rates of side effects differ”.*

**Reviewer #3:**

3) Page 4 Line 87: Would reword - This review is a comprehensive report on the prevalence of dermatological toxicity in patients ...

**Our response:** As suggested by the reviewer, we reworded the sentence: *“This review is a comprehensive report on the prevalence of dermatological toxicity in patients with melanoma using ICIs as monotherapy and/or in combination with chemotherapy and/or radiotherapy.”*

**Reviewer #3:**

4) Table 1: I would still further simplify. Would remove the Duration of treatment, Follow up, and main conclusions since really just highlighting incidence of cutaneous irAE

**Our response:** We removed the follow-up column from table 1. The duration of treatment and main conclusion columns had already been removed on previous revision. The table 1 now presents the following data: author/year/country, study design, sample size, age, drug (dose and schedule) and dermatological toxicities.

**Reviewer #3:**

5) Page 29 Line 329: Reword - Cutaneous AEs are often the first toxicities

**Our response:** We reworded as suggested: *“Cutaneous AEs are often the first toxicities to occur with the use of ICIs. (…)”*

**Reviewer #3:**

6) Page 29 Line 330: Reword - ... these toxicities may lead to IDI dose interruption or treatment discontinuation (there really isn't dose reduction with ICIs)

**Our response:** As suggested by the reviewer, we reworded the sentence: *“Despite being self-limited, these toxicities may lead to ICI dose interruption or treatment discontinuation.”*

**Reviewer #3:**

7) Page 32 Line 410: Would take sentence that starts with "Although it is possible ... and make it last sentence of conclusion

Our response: Done.

**Reviewer #3:**

8) Page 33 Line 424: Would remove sentence that starts with "Vitiligo" and "Regarding" Make conclusion one paragraph

Our response: We removed the sentences as suggested by the reviewer, included the sentence as suggested by the reviewer’s comment #7, and made the conclusion one paragraph as follow: *“The results of this review and meta-analysis show that the most prevalent irAEs are pruritus and rash. Even though mild and moderate irAEs were reported more frequently than severe irAEs in the included studies, there was also a significant representation of more severe AEs. Grade 3 or 4 irAEs have been associated with the use of ipilimumab. Although it is possible to manage these AEs in most cases, early identification plays a key role in the prevention of severe cases, avoiding treatment interruption.”*
